# Using Personalized Anchors to Establish Routine Meditation Practice With a Mobile App: Randomized Controlled Trial

**DOI:** 10.2196/32794

**Published:** 2021-12-22

**Authors:** Chad Stecher, Mariah Sullivan, Jennifer Huberty

**Affiliations:** 1 College of Health Solutions Arizona State University Phoenix, AZ United States

**Keywords:** mindfulness, meditation, mobile meditation app, behavioral persistence, habit formation, randomized controlled trial, mental health, physical health, app engagement, routine

## Abstract

**Background:**

Physical and mental health benefits can be attained from persistent, long-term performance of mindfulness meditation with a mobile meditation app, but in general, few mobile health app users persistently engage at a level necessary to attain the corresponding health benefits. Anchoring or pairing meditation with a mobile app to an existing daily routine can establish an unconsciously initiated meditation routine that may improve meditation persistence.

**Objective:**

The purpose of this study was to test the use of either personalized anchors or fixed anchors for establishing a persistent meditation app routine with the mobile app, Calm.

**Methods:**

We conducted a randomized controlled trial and randomly assigned participants to one of 3 study groups: (1) a personalized anchor (PA) group, (2) fixed anchor (FA) group, or (3) control group that did not use the anchoring strategy. All participants received app-delivered reminder messages to meditate for at least 10 minutes a day using the Calm app for an 8-week intervention period, and app usage data continued to be collected for an additional 8-week follow-up period to measure meditation persistence. Baseline, week 8, and week 16 surveys were administered to assess demographics, socioeconomic status, and changes in self-reported habit strength.

**Results:**

A total of 101 participants across the 3 study groups were included in the final analysis: (1) PA (n=56), (2) FA (n=49), and (3) control group (n=62). Participants were predominantly White (83/101, 82.2%), female (77/101, 76.2%), and college educated (ie, bachelor’s or graduate degree; 82/101, 81.2%). The FA group had a significantly higher average odds of daily meditation during the intervention (1.14 odds ratio [OR]; 95% CI 1.02-1.33; *P*=.04), and all participants experienced a linear decline in their odds of daily meditation during the 8-week intervention (0.96 OR; 95% CI 0.95-0.96; *P*<.001). Importantly, the FA group showed a significantly smaller decline in the linear trend of their odds of daily meditation during the 8-week follow-up (their daily trend increased by 1.04 OR from their trend during the intervention; 95% CI 1.01-1.06; *P*=.03). Additionally, those who more frequently adhered to their anchoring strategy during the intervention typically used anchors that occurred in the morning and showed a significantly smaller decline in their odds of daily meditation during the 8-week follow-up period (1.13 OR; 95% CI 1.02-1.35; *P*=.007).

**Conclusions:**

The FA group had more persistent meditation with the app, but participants in the FA or PA groups who more frequently adhered to their anchoring strategy during the intervention had the most persistent meditation routines, and almost all of these high anchorers used morning anchors. These findings suggest that the anchoring strategy can create persistent meditation routines with a mobile app. However, future studies should combine anchoring with additional intervention tools (eg, incentives) to help more participants successfully establish an anchored meditation routine.

**Trial Registration:**

ClinicalTrials.gov NCT04378530; https://clinicaltrials.gov/ct2/show/NCT04378530

## Introduction

Mindfulness meditation is an evidence-based health behavior regimen that can produce a wide range of physical and mental health benefits, such as reduced blood pressure and decreased symptoms of anxiety, depression, and insomnia [1-3]. However, similar to other health behaviors, such as physical activity, the benefits of mindfulness meditation are primarily experienced after persistent, long-term performance [4-7]. Research has shown that increases in meditation frequency, duration, and long-term performance are all associated with greater health benefits among both clinical and general populations of adults [8-10]. Additionally, many of the proposed mechanisms for the benefits of meditation include biological changes, such as altering brain morphology, which happen over time through persistent meditation performance [11,12].

A diverse set of barriers exist to persistently performing mindfulness meditation, including structural (eg, financial and access-related)-, social (eg, stigma and peer-support)-, and individual (eg, impatience and motivation)-level factors. These multifaceted barriers have been shown to inhibit persistent mindfulness meditation practices and the persistent performance of other health-promoting behaviors [13-16], and thus novel behavioral interventions are still needed to help individuals attain the benefits from the long-term performance of healthy behaviors.

Mindfulness meditation has been successfully adapted for mobile phone apps, which helps to address several of the common structural and social barriers to persistent meditation. Mindfulness meditation apps are easily accessible, scalable, and cost-effective, improving individuals’ access to meditation instruction and education [17,18]. Numerous commercial meditation apps are available to the public, and to date, the 2 leading apps are Headspace and Calm with 65 and 200 million downloads, respectively [3,19,20]. Although access to these popular apps is not free (roughly US $70 for an annual subscription), the cost is significantly lower than that of in-person, guided meditations. Additionally, employers are increasingly providing free access to meditation apps to their employees to help them improve their mental health and workplace productivity [18].

Interventions using commercial meditation apps have proven to be feasible and have demonstrated small- to medium-sized effects in reducing symptoms of depression and anxiety and increasing life satisfaction and positive affect [17,21]. Despite the accessibility and popularity of commercial meditation apps, app-based meditation persistence rates are low [22-24]. For example, a recent review found that adherence to app-based meditation interventions can be as low as 24% [25], and in the real world (ie, not in a research study), only 2% of health app users persistently engage at a level necessary to attain the corresponding health benefits [25-27].

Although mindfulness meditation apps have addressed several important structural and social barriers, the low persistence among app users might result from a lack of successful strategies for overcoming common individual-level barriers (eg, impatience and motivation) to persistent meditation. Behavioral economics and psychology research has demonstrated that individual-level barriers are significant determinants of nonpersistent (ie, only short-term) health behavior change, even after structural and social barriers have been overcome [13,28-30]. This has also been documented in the mobile health app literature: despite the popularity and ability of meditation apps to improve mental health, sustained engagement among mobile health app subscribers is low [4,24,25,31]. Moreover, a recent systematic review and meta-analysis of mental health app interventions reported app participation consistently decreased over time [4]. Therefore, novel strategies are needed to address individual-level barriers and help individuals increase and maintain their use of mindfulness meditation apps.

One strategy for overcoming individual-level barriers to mindfulness meditation app use may be the development of a meditation routine. Psychology research has shown that behaviors consistently performed in response to the same contextual (or environmental) cue become routinized, meaning they are completed with little or no cognitive effort [32,33]. One successful strategy for establishing a new routine is anchoring or pairing the new behavior to an existing routine that is already executed with very little cognitive effort [34-36]. For example, one might pair his or her daily meditation with their existing routine of an afternoon walk in order to routinize an afternoon meditation practice. Existing anchoring interventions have successfully established these reflexive or automatic routines for smoking cessation [37] and medication adherence [38,39]. However, the success of anchoring interventions has so far been limited to simple behaviors, such as drinking water or taking medications. Additionally, anchoring has largely only been effective for participants with high initial intrinsic motivation [40-44], so it is still unknown whether anchoring can help an individual successfully establish a persistent meditation app routine.

Furthermore, there are important design considerations in anchoring interventions that have not been rigorously tested in the literature, such as how to optimally select a participant’s anchor. Research has shown that personalization is an important component to many other health interventions [45-51]; however, the theory of contextually cued routines is new for most people, so it may be difficult for participants to identify their own (ie, personalized) existing routine that can serve as an effective anchor for a new meditation routine. It has also been shown that daily routines most frequently occur in the morning [44,52], and recent research on circadian rhythms has suggested that routinization may be easier in the morning [53]. Thus, the purpose of this study was to test the efficacy of using a personalized anchor versus having an anchor assigned in the morning (ie, fixed) for successfully establishing a persistent meditation app routine using the mobile app Calm. These 2 intervention groups (ie, personalized vs fixed anchors) received app-delivered reminder messages of their anchoring strategy for an 8-week period, and the persistence of the meditation routine over the subsequent 8 weeks was compared between these 2 groups and a control group that did not use the anchoring strategy for daily meditation. We hypothesized that the personalized anchor group would be the most persistent over the 8-week follow-up period and that both intervention groups would have significantly greater meditation persistence relative to the control group.

## Methods

### Recruitment

A randomized controlled trial was conducted between July 2020 and March 2021 with an 8-week intervention period, an 8-week follow-up period, and survey assessments at baseline, week 8, and week 16. The Institutional Review Board at Arizona State University approved this study (STUDY00011788), and all participants provided consent electronically prior to participating in the survey. This study design was preregistered on ClinicalTrials.gov (NCT04378530) and was funded by Arizona State University. The CONSORT file is available in Multimedia Appendix 1.

Study recruitment took place from July 2020 to August 2020. Participants were paying subscribers to the Calm app who were identified as not having already formed a daily meditation routine. Specifically, subscribers were eligible if they had subscribed to the Calm app after January 2020, had not completed a meditation session with the app in the past 30 days, and did not report practicing meditation with or without the app for more than 60 minutes in 1 month over the past 6 months. Additionally, new subscribers were eligible if they could read and understand English, were willing to be randomized, and were between 18 and 60 years old (see Textbox 1 for a full list of study eligibility criteria). Eligible subscribers were identified by Calm and invited to participate in the study via email. The email contained a brief overview of the study and a link to a short eligibility survey, and Qualtrics software was used to verify that participants satisfied all remaining study eligibility criteria. Eligible participants were then automatically directed to read and electronically sign an informed consent document in Qualtrics. Consenting participants were then contacted by the research team via email to complete the baseline questionnaire in Qualtrics. Once they completed the questionnaire, participants were randomized to 1 of 3 study groups using a predetermined allocation list generated on Randomizer.org by a researcher not involved in the participant assignment. Participants were then assigned to a study group based on the allocation list and the order in which they were enrolled in the study.

**Eligibility criteria.**

**Inclusion criteria**

• 18-60 years of age

• Purchased Calm after January 2020

• Inactive: have not used app in the past 30 days

• Own an iOS/Android smartphone

• Own home internet or unlimited data plan

• Able to read and understand English

• Willing to be randomized

**Exclusion criteria**

• Report practicing mindfulness meditation >60 min in 1 month within the last 6 months

• Any meditation sessions with app in the past last 30 days

• Currently reside outside the USA

### Intervention

Participants were randomized into a personalized anchor (PA) group, fixed anchor (FA) group, or control group (CG). Participants in this study used their own paid Calm accounts to access the app during the study. After completing the baseline survey, participants were sent a link to watch an instructional video that provided information about the benefits of meditating 10 minutes per day and study group–specific instructions on how to participate in the study. For those in the PA group, the video instructed participants to select an existing routine to which they would anchor their 10 minutes of daily meditation practice. The PA group’s instructional video emphasized the importance of selecting a consistently occurring daily routine that could reliably be followed by 10 or more minutes of meditation and provided clear examples of such existing routines (eg, “After I finish my coffee in the afternoon” or “After I finish breakfast in the morning”). For those in the FA group, participants were instructed to use a fixed anchor provided by the research team to which they would anchor their 10 minutes of daily meditation practice. The anchor provided was the following: “After I finish in the bathroom (brushing teeth, removing mouth guard, etc.) in the morning, I will meditate for at least 10 minutes.” Participants in the CG were given information about the mental health benefits of meditating for at least 10 minutes per day and instructed to complete 10 minutes of daily meditation but were not given any instruction on how or when to meditate. Participants were blinded to the other intervention protocols and did not know what intervention component was the focus of this study. To verify participants’ comprehension of their study group–specific instructions, participants completed a 3-question comprehension quiz in Qualtrics and were given unlimited chances to answer each question correctly. Once all questions were correctly answered, participants were emailed with a start date for their intervention and they were provided with a written copy of the study instructions.

During the 8-week intervention period, all participants received a daily app–delivered reminder message (ie, push notification) to either meditate for at least 10 minutes or to meditate for at least 10 minutes using their anchor. Messages were randomly delivered at either 8 AM, 1 PM, or 6 PM (ie, a 33.3% chance of receiving the daily message at 1 of the 3 possible times), with adjustments made for participants’ time zone. The message content was also randomized with a 50% chance of receiving 1 of 2 message types. The first message type included study group–specific reminders reinforcing participants’ use of either their personalized or fixed anchors, or reminding the control group to meditate. The second message type was evenly randomized between reminders to use 3 motivational tools in the Calm app: mood check-ins, the meditation activity tracker, or the in-app daily reminder tool. The success of each type, timing, and sequence of daily supports was evaluated based on both participants’ daily app usage data and ecological momentary assessments collected via SMS text messages once per evening (8 PM) during the 8-week intervention. The results from this microrandomized trial on the effectiveness of different daily reminder messages are not reported in this paper, and it is important to note that this microrandomized trial study design meant that each message type, timing, and sequence were randomly delivered across all study groups; thus, the sequence of messages would not bias our analysis of the overall study group differences in meditation persistence during this study.

Participants were initially instructed to use their anchors (PA and FA groups) and meditate for 10 minutes per day (all groups) for 8 weeks. After 8 weeks, participants were emailed a postintervention survey to complete and were encouraged to continue meditating but were not given further instructions. Participants were emailed again at the end of the 8-week follow-up period and given a final questionnaire to complete.

### Surveys

The baseline, postintervention, and final questionnaires were all completed in Qualtrics. Participants were asked to respond using “A little bit,” “Neutral,” “Quite a bit,” or “A lot” to the following 3 questions about the COVID-19 pandemic: “To what extent do you feel the COVID-19 pandemic has affected your mental health?”, “To what extent do you feel the COVID-19 pandemic has affected your physical health?”, and “To what extent do you feel the COVID-19 pandemic has affected your stress?” Participants also completed the Self-Report Behavioral Automaticity Index (SRBAI) on each survey to assess the strength of their meditation habit (ie, self-reported habit strength) [54]. The SRBAI contains 4 items scored on a 5-point Likert scale from 1 “Strongly disagree” to 5 “Strongly agree” in response to statements like “Daily meditation is something I do automatically,” where a higher sum of item scores indicates a stronger habit. The SRBAI has a Cronbach’s α of ≥.81 and was designed using discriminant content validity while preserving strong predictive validity [54]. Each survey also asked participants to rate their overall health as either “Poor,” “Fair,” “Good,” “Very Good,” or “Excellent.” On the baseline survey, participants answered questions on their demographic and socioeconomic characteristics.

### Outcomes

The primary outcome measure for this study was a binary measure of any daily meditation over the 16-week study, which was derived from participants’ Calm app usage data provided by the Calm analytics team. Specifically, we used minute-level data on the time of day and duration of meditation sessions with the Calm app to construct an indicator variable equal to 1 if a participant completed any minutes of meditation on a given day, and 0 otherwise. To study how our intervention impacted meditation persistence, we examined how the odds of performing any daily meditation changed over time both during and after the intervention. The app usage data were also used to construct an indicator variable equal to 1 if a participant completed any minutes of meditation within 1 hour of the typical time that their personalized anchor was reported to occur (this typical time was collected when the PA group selected their anchor) or when the fixed anchor was expected to occur (8 AM). This measure of temporally consistent meditation was used to study participants’ adherence to their anchoring strategy during and after the intervention. The secondary outcome of interest was the change in SRBAI between the study groups.

### Statistical Analysis

A total sample size of 150 participants (study group sizes of 50) was targeted based on our available resources, and our expected statistical power was informed by prior interventions using the Calm app [3,55,56]. Assuming a small-to-medium-effect size of 0.20, study group sizes of 50 yielded a statistical power of 1–β =.76 for detecting study group x day–level differences in linear models of our repeated daily outcome (any meditation minutes) over the 16-week study at α=.05 (calculated using GLIMMPSE [57]).

Participants’ demographic, socioeconomic, and health characteristics were compared across the 3 study groups to confirm that the randomization was effective using the Kruskal-Wallis nonparametric tests of equality (Table 1).

The primary outcome measuring the odds of any daily meditation was analyzed using panel logistic regression models with participant-level random effects. Aggregate study group differences in the primary outcome were estimated using separate indicator variables for the PA and FA groups, where the CG was the omitted reference group, and differences in the primary outcome over time were estimated using interaction terms between each study group indicator variable and a daily time trend. Two modeling approaches for the daily time trend were used: (1) a single linear time trend over the full 16-week study and (2) a piecewise linear trend with a breakpoint after the 8-week intervention (ie, daily reminder messages) being withdrawn. The same panel logistic model with random effects was estimated for an outcome variable indicating whether participants performed any minutes of meditation within 1 hour of the expected time of their anchor (referred to as “anchored meditations”). These models were estimated as intention-to-treat analyses that used daily Calm app data for all participants who were retained in the study.

**Table 1:** Participant characteristics by study group.

Characteristic	Control, n (%) (N=37)	Fixed anchor, n (%) (N=27)	Personalized anchor, n (%) (N=37)	Two-sided *P* value^a^
Black	0 (0.00)	1 (3.70)	3 (8.11)	.21
Asian/Arab	2 (5.41)	1 (3.70)	1 (2.70)	.84
White	33 (89.19)	22 (81.48)	28 (75.68)	.32
Bi- or multiracial	0 (0.00)	1 (3.70)	1 (2.70)	.54
Race: nonresponse	2 (5.41)	0 (0.00)	2 (5.41)	.47
Male	8 (21.62)	6 (22.22)	4 (10.81)	.38
Female	28 (75.68)	19 (70.37)	30 (81.08)	.61
Less than 20 k^b^	0 (0.00)	1 (3.70)	3 (8.11)	.20
21-40 k^b^	2 (5.41)	1 (3.70)	6 (16.22)	.15
41-60 k^b^	5 (13.51)	5 (18.52)	7 (18.92)	.80
61-80 k^b^	2 (5.41)	2 (7.41)	1 (2.70)	.69
81-100 k^b^	8 (21.62)	8 (29.63)	1 (2.70)	.01
More than 100 k^b^	19 (51.35)	10 (37.04)	17 (45.95)	.53
Married	26 (70.27)	12 (44.44)	17 (45.95)	.05
Partnered	2 (5.41)	6 (22.22)	3 (8.11)	.08
Single/divorced/widowed	9 (24.32)	9 (33.33)	17 (45.95)	.15
Graduate degree	24 (64.86)	15 (55.56)	15 (40.54)	.11
Bachelor's degree	6 (16.22)	10 (37.04)	12 (32.43)	.14
Less than a bachelor’s	7 (18.92)	2 (7.41)	10 (27.03)	.14
Poor health	0 (0.00)	4 (14.81)	1 (2.70)	.02
Fair health	7 (18.92)	6 (22.22)	9 (24.32)	.85
Good health	12 (32.43)	10 (37.04)	13 (35.14)	.93
Very good health	13 (35.14)	4 (14.81)	12 (32.43)	.17
Excellent health	4 (10.81)	3 (11.11)	0 (0.00)	.12
Currently with depression	11 (29.73)	9 (33.33)	11 (29.73)	.94
COVID-19 stress	30 (83.33)	20 (74.07)	27 (77.14)	.66
COVID-19 mental health	24 (66.67)	21 (77.78)	24 (68.57)	.61
COVID-19 physical health	15 (41.67)	12 (44.44)	12 (34.29)	.69

^a^Two-sided *P* values are presented for Kruskal-Wallis nonparametric tests of equality for each measure of participants’ characteristics across the 3 study groups.

^b^Income in US $.

In subgroup analyses, participants were split into high- and low-meditation subgroups based on their total number of days with any meditation during the 8-week intervention. The high-meditation subgroup was defined as those participants who meditated on 14 (the median number of days) or more of the intervention days. All other participants were placed in the low-meditation subgroup. These subgroups were created to test whether the success of the anchoring strategy differed based on the total number of meditations performed during the intervention. Participants in the PA and FA groups were also split according to the number of intervention days that they potentially meditated with the Calm app using their anchor during the intervention. Participants from the PA and FA groups were classified as high anchorers if they completed 12 (the median number of days using one’s anchor) or more meditations within 1 hour of the expected time of their anchor. All other participants in the PA and FA groups were considered low anchorers, and the CG did not use the anchoring strategy and so were not classified as either high or low anchorers. These additional subgroups were created to examine how the success of the anchoring strategy varied based on the number of anchored meditations during the intervention.

Study group differences in the SRBAI between the baseline and postintervention survey were analyzed using analysis of variance (ANOVA), and pairwise comparisons between the PA and FA groups and the CG used were analyzed with the *t* test. All statistical analyses were performed using Stata/MP (StataCorp) 16.1 for Windows. (Microsoft Corp).

## Results

**Figure 1:**
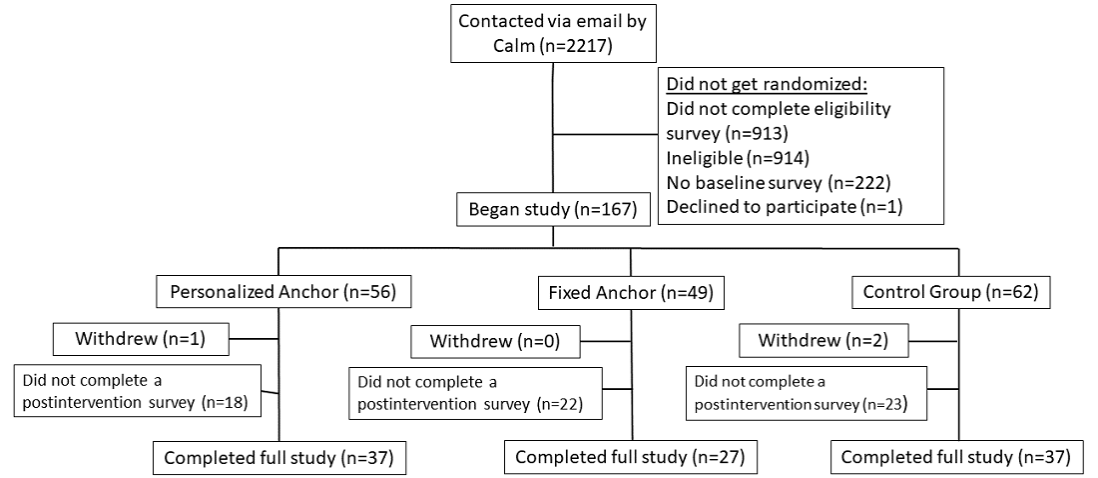
Study flow diagram of participant enrollment and randomization.

A total of 2217 Calm subscribers were emailed to participate in this study. Among those who completed the eligibility survey and were identified as eligible, 167 provided informed consent, completed the baseline survey, and were randomized into 1 of the 3 study groups: (1) the PA group (n=56), (2) the FA group (n=49), or the CG (n=62). Figure 1 is a flow diagram outlining participant enrollment, randomization, and retention.

After a few participants asked to withdraw (n=3), a total of 101 participants completed at least 1 postintervention survey (either week 8 or week 16) and were included in the final analysis. Due to the different attrition rates across study groups, the final analytical sample was not balanced in size across groups, limiting the statistical power of our analyses. However, Table 1 shows that the study groups were still balanced on most of the observed participant characteristics. Participants were predominantly White (83/101, 82.2%), female (77/101, 76.2%), college educated (ie, bachelor’s or graduate degree; 82/101, 81.2%), and earned $81,000 per year and above (63/101, 62.4%). Additionally, only 26.7% (27/101) of participants reported “Poor” or “Fair” health, 76.2% (77/101) reported that COVID-19 has affected their stress either “Quite a bit” or “A lot,” and 68.3% (69/101) reported that COVID-19 has affected their mental health either “Quite a bit” or “A lot.” Importantly, there were few statistically significant differences between study groups at baseline (see Table 1). The only observable differences between study groups were in terms of marital status and the percent reporting “poor” health, where the 2 treatment groups (PA and FA) were less likely to be married and more likely to report poor health than was the control group. Given these differences across study groups, we included covariates for each of these characteristics in additional regression models presented in Multimedia Appendix 2.

**Figure 2:**
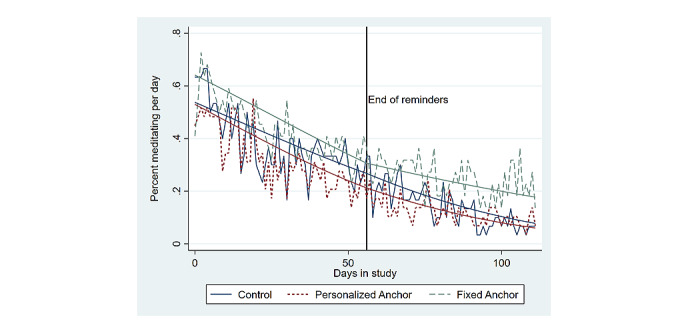
Daily percent of participants who performed any minutes of meditation.

Table 2 displays the study group differences in the daily odds of any meditation (our primary outcome). Specifically, Table 2 shows the exponentiated coefficients from panel logistic regression models estimated with participant-level random effects predicting the primary outcome among the full sample (column 1) and separately estimated among the high-meditation subgroup (column 2). The FA group had a significantly higher average odds of daily meditation during the intervention (1.14 odds ratio [OR]; 95% CI 1.02-1.33; *P*=.04), and all participants experienced a significant linear decline in their odds of daily meditation during the 8-week intervention (0.96 OR; 95% CI 0.95-0.96; *P*<.001). Additionally, the FA group showed a significantly smaller decline in the linear trend of their odds of daily meditation during the 8-week follow-up period (their daily trend increased by 1.04 OR from their trend during the intervention; 95% CI 1.01-1.06; *P*=.03 during the follow-up). A separate model was estimated that also included measures of participants’ race, gender, education, marital status, health status, and an identifier for self-reporting being depressed, and these results are presented in Multimedia Appendix 2 and do not significantly differ from the model without these additional participant characteristics. To visualize these study group differences in our primary outcome, Figure 2 displays both the raw and predicted daily probability of any minutes of meditation for each study group based on the coefficient estimates from the full analytic sample shown in column 1 of Table 2.

**Table 2:** Treatment effects on the odds of daily meditation.

Independent variables:	All participants, OR^a^ (95% CI)	High-meditation subgroup, OR (95% CI)
Fixed anchor	1.139 (1.019-1.326)^**^	1.081 (1.021-1.310)^**^
Personalized anchor	1.012 (0.270-3.860)	1.062 (0.884-1.276)
Days in study	0.960 (0.956-0.964)^***^	0.964 (0.957-0.970)^***^
Fixed anchor × days	0.989 (0.977-1.000)	1.001 (0.999-1.003)
Personalized anchor × days	0.993 (0.976-1.002)	0.987 (0.974-1.000) ^*^
Days postintervention	0.992 (0.978-1.006)	0.985 (0.969-1.000) ^*^
Fixed anchor × postintervention days	1.035 (1.013-1.057) ^***^	1.005 (0.978-1.032)
Personalized anchor × postintervention days	1.013 (0.990-1.036)	0.985 (0.975-1.000)^*^
Participant-day observations, n^b^	11,312	5712
Participants, n^b^	101	51

^a^OR: odds ratio.

^b^Data in this row are expressed as integers and not odds ratio and CI.

^*^P<.10.

^**^P<.05.

^***^P<.01.

**Figure 3:**
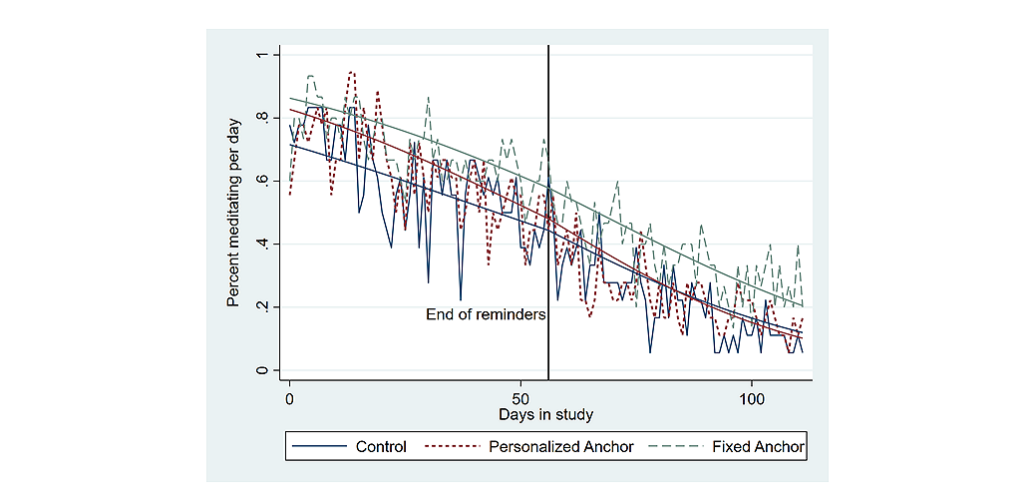
Daily percent of participants in the high-meditation subgroup who performed any minutes of meditation.

To examine if the anchoring strategy was more successful for more frequent meditators, the raw and predicted daily probability of any minutes of meditation among the high-meditation subgroup (n=51) in each study group was determined (Figure 3). The corresponding regression results in Table 2 show that among the high-meditation subgroup, the FA group still had a significantly higher average odds of daily meditation during the intervention (1.08 OR; 95% CI 1.02-1.31; *P*=.03), and all participants experienced a significant linear decline in their odds of daily meditation during the 8-week intervention (0.96 OR; 95% CI 0.96-0.97; *P*<.001). However, there was no statistically significant difference between study groups in the decline of daily odds of meditation during the 8-week follow-up among the high-meditation subgroup.

**Figure 4:**
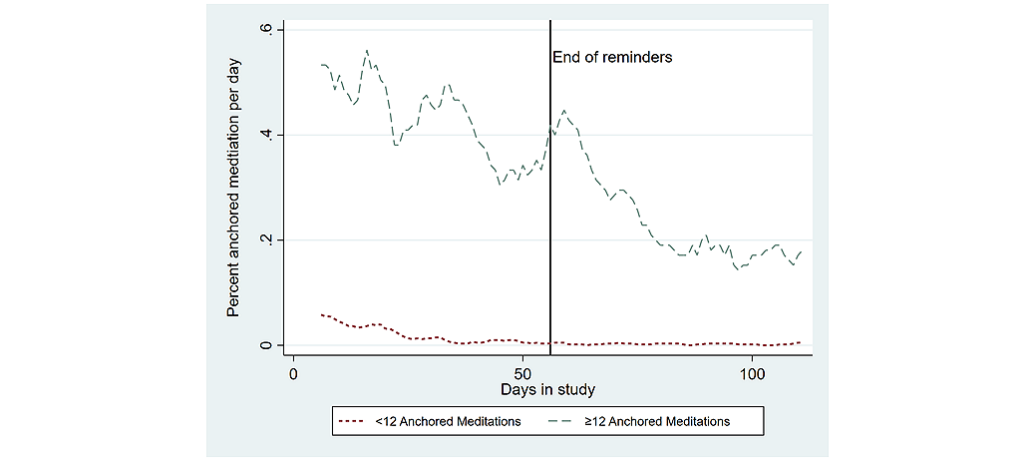
Daily percent of participants who performed any minutes of anchored meditation.

To visualize how the anchoring strategy impacted meditation persistence, Figure 4 plots the average daily percent of participants who completed any minutes of meditation within 1 hour of the expected time of their anchor (ie, anchored meditations) among the high anchorers (n=19) and separately among the low anchorers (n=45). The high-anchorer subgroup was composed of 13 participants from the FA group and 6 participants from the PA group, which demonstrates the relative success of using the fixed morning anchor versus allowing participants to select their own anchor. Additionally, 4 out of the 6 high anchorers from the PA group selected a morning anchor that occurred between 7 AM and 9 AM, which further suggests that morning anchors are the most likely to be successful. The trends in Figure 4 show that most participants (ie, the low anchorers) did not use their anchoring strategy beyond the first 4 weeks of the intervention but that anchored meditations remained fairly persistent among the high anchorers.

**Figure 5:**
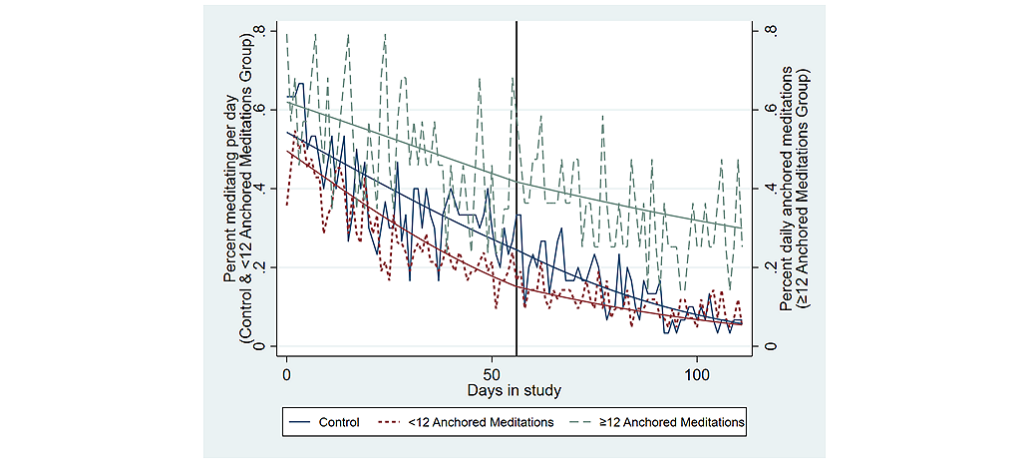
Daily percent of participants who performed any minutes of meditation or any minutes of anchored meditations.

The stronger persistence in anchored meditations among the high anchorers was tested empirically and is shown in Table 3, which displays the panel logistic regression results from models predicting the odds of any minutes of meditation for the low anchorers and those in the CG or the odds of any anchored meditations among the high anchorers. This split outcome variable provided a more conservative test of the differences in meditation persistence between the high anchorers versus the low anchorers or the CG because all nonanchored meditations were not considered as evidence of meditation persistence for the high anchorers. The high anchorers had a significantly higher average odds of daily meditation during the intervention (34.68 OR; 95% CI 5.70-210.80; *P*=.008), and all participants experienced a significant linear decline in their odds of daily meditation during the 8-week intervention (0.96 OR; 95% CI 0.96-0.97; *P*<.001). Importantly, the high anchorers showed a significantly smaller decline in the linear trend of their odds of daily meditation during the 8-week follow-up period (their daily trend increased by 1.13 OR from their trend during the intervention; 95% CI 1.02-1.35; *P*=.007 during the follow-up). A separate model was estimated for this split outcome that also included measures of participants’ race, gender, education, marital status, health status, and an identifier for self-reporting being depressed, and these results are presented in Multimedia Appendix 2 and do not significantly differ from the model without these additional participant characteristics. Figure 5 displays the raw and predicted probability of this split outcome for high anchorers, low anchorers, and the CG.

**Table 3:** Effect of successfully anchoring on the odds of daily meditation.

Independent variables	Linear time trend, OR^a^ (95% CI)	Piecewise linear trend, OR (95% CI)
<12 anchored meditations	0.613 (0.206-1.824)	0.793 (0.264-2.388)
≥12 anchored meditations	28.079 (4.773-165.201) ^***^	34.675 (5.704-210.796)^***^
Days in study	0.960 (0.956-0.964)^***^	0.964 (0.957-0.971)^***^
<12 anchored meditations × days	0.998 (0.993-1.004)	0.987 (0.977-1.002)
≥12 anchored meditations × days	1.002 (0.994-1.010)	0.994 (0.979-1.009)
Days postintervention	—^b^	0.992 (0.978-1.006)
<12 anchored meditations × postintervention days	—	1.067 (0.990-1.145)
≥12 anchored meditations × postintervention days	—	1.129 (1.019-1.351)^***^
Participant-day observations, n^c^	11,312	11,312
Participants, n^c^	101	101

^a^OR: odds ratio.

^b^Not included in the model.

^c^Data in this row are expressed as integers and not odds ratio and CI.

^*^P<.10.

^**^P<.05.

^***^P<.01.

**Figure 6:**
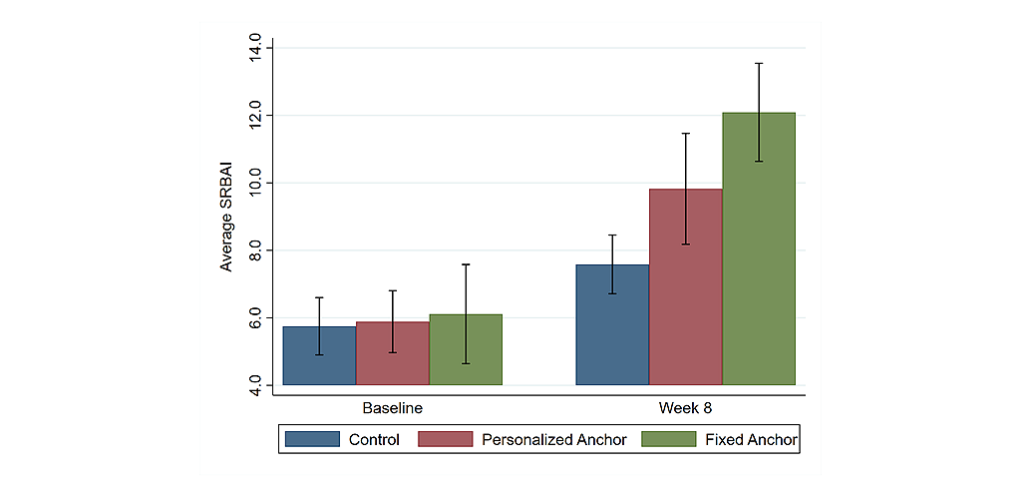
Average Self-Reported Behavioral Automaticity Index. SRBAI: Self-Reported Behavioral Automaticity Index.

Figure 6 displays the average self-reported meditation habit strength, our secondary outcome, among the 3 study groups on the baseline and week 8 surveys. There was no statistically significant difference in self-reported habit strength between the study groups at baseline. Participants in the FA group reported a significantly higher increase in self-reported habit strength between baseline and week 8 than did the CG (4.56 greater SRBAI increase; 95% CI 1.46-7.66; *P*<.001), while the differences between the FA and PA groups and PA and CG were not statistically significant.

## Discussion

### Principal Findings

This study tested the efficacy of using either PAs or FAs for establishing a persistent meditation app routine with the mobile app, Calm. Although the results found that all study groups (ie, PA, FA, and CG) experienced an equal decline in their daily odds of performing any minutes of meditation with the Calm app during the 8-week intervention, the FA group was significantly more persistent (ie, smaller daily decline in the odds of any meditation) during the 8-week follow-up period. Subgroup analyses revealed that performing a larger number of meditations during the intervention was not sufficient for displaying meditation persistence. Instead, the participants who were high anchorers during the intervention (ie, equal to or above the median number of meditations performed within 1 hour of the expected time of their anchor) showed the most persistent meditation routines during the follow-up period. These findings indicate that the anchoring strategy can create persistent meditation routines for some participants but that additional intervention tools are likely needed to help more participants successfully adhere to their anchored meditation routine.

The results in Table 2 and Figure 3 show that simply performing meditation on more days of the intervention was not associated with higher meditation persistence among any of the 3 study groups. Specifically, Figure 3 shows that despite high meditators (ie, participants who meditated on 14—the median number of days—or more of the intervention days) having an average daily probability of meditating roughly equal to 80% at the start of the study, the average high meditator in any of the 3 study groups displayed a steady decline in their daily probability of meditating. This observation stands in contrast to the expected role that high behavioral performance should have on behavioral persistence according to standard microeconomic theory of habit formation [58,59]. As high-meditators’ level of meditation performance did not sufficiently increase their marginal utility for continuing their meditation behavior, the results suggest that meditation needs to be performed for a longer duration of time (ie, more days with any meditation) in order to form a persistent routine or that alternative theories of habit formation may be more appropriate for understanding persistent meditation routines.

Additionally, the results show that high anchorers were significantly more persistent in their daily meditation, and these findings were estimated using only the anchored meditations to measure persistence among the high-anchorer subgroup. In other words, high anchorers were not just more likely to meditate at any time of day, but this subgroup was more likely to meditate at a time that corresponded to their anchor than the control group or the low anchorers were to meditate at any time of day. This observation supports the theory that contextually cueing behaviors is one method for creating a persistent meditation routine [32,33]. As fewer than half (19/64, 30%) of the participants in either the FA or PA groups were high anchorers, these results also suggest that setting an anchoring strategy and receiving app-delivered reminder messages are not sufficient for helping all participants adhere to their anchoring strategy. Importantly, almost all of the high anchorers were participants who either selected a morning anchor or were given the fixed morning anchor, which indicates that meditating in the morning might be an important strategy for establishing persistent meditation routines and warrants further research.

The results from the SRBAI (ie, self-reported habit strength) show that those in the FA group experienced the largest increase in habit strength. However, there was an increase in self-reported habit strength among all study groups, including the CG. Additionally, self-reported habit strength increased on average for all study groups despite the clear decline in daily meditation performance observed in the objective app usage data. These trends highlight a potential limitation of this self-report habit strength measure: since habitual behaviors are theorized to be unconsciously initiated, individuals should not be able to recall their experience performing the behavior (in this case meditation). Thus, this measure may be capturing participants’ perceived self-efficacy or fluency for meditation [60], which suggests that simply being involved in a study and receiving information on the benefits of meditating daily for 10 or more minutes might have boosted participants’ feeling of behavioral competence or self-efficacy for meditation. Therefore, although this self-reported habit strength was significantly greater among the FA participants who anchored meditation in the morning, these results should be interpreted with caution.

### Prior Work

This study contributes to the existing literature testing anchoring interventions for health behaviors, which has already demonstrated the success of anchoring for establishing persistent smoking cessation routines [37,61] and medication adherence routines [38,39]. However, the anchoring approach has been less effective in other settings, such as demonstrating limited efficacy for improving diets [40,62,63], which suggests that the success of anchoring may vary depending on the behavioral complexity of the targeted new routine. Our study shows that anchoring can help to improve the persistence of meditation with a mobile app for some participants, but the success of anchoring was not universally experienced by all participants, and further research is needed to determine whether anchoring can be more effectively implemented to establish persistent meditation routines with a mobile app.

It is important to note that our design of the anchoring intervention was targeted toward establishing “instigation” habits as opposed to “execution” habits for daily meditation [64]. In other words, the suggested anchors were all chosen to help initiate meditation with the Calm app as opposed to helping participants continue to perform a given meditation session. This was because we hypothesized that continuing to perform a given meditation session is a relatively easier action since meditation is generally a passive behavior and most of the meditations with the app are timed, so users do not need to self-monitor the clock and their time meditating. Future studies should test the efficacy of anchoring interventions that target the execution component of daily meditation, which may help us understand how anchoring can be successfully applied to complex behaviors like daily meditation.

Finally, this study demonstrates that an 8-week intervention was not sufficiently long for even the high anchorers to form a meditation routine. Existing research has suggested that it takes anywhere from 18 to 254 days to successfully form a new routine [65], so our results help to increase the lower bound on this range for meditation routines. Additional research is needed to generate a more precise estimate of the average number of days of behavioral performance for successfully routinizing meditation with a mobile app.

### Limitations

Although this was the first study to use personalized or fixed anchors for establishing a persistent meditation app routine with a consumer-based app (ie, Calm) and there were no unexpected events, there were still a number of limitations. First, we had a homogeneous, small sample size limiting the generalizability of our findings, particularly to other racial groups and people of different socioeconomic status. Second, our study targeted dormant users of Calm who had paid for an annual subscription but had not recently used the app, which again limits the generalizability of our results for other types of app users. Third, the daily app–delivered reminder messages appeared to be an ineffective method of boosting most participants’ attention to and use of the anchoring strategy, so it is difficult to know whether a longer duration of intervention or increased intervention supports are necessary to increase adherence to the anchoring strategy and more rigorously test the efficacy of this intervention approach for establishing behavioral routines. Finally, a significant degree of study attrition from either withdrawals or missing survey data occurred during the intervention, which limited the statistical power of our analyses.

**Conclusions**

This study tested the efficacy of using either personalized or fixed anchors for establishing a persistent meditation app routine with the mobile app Calm. Participants given the FA of meditating in the morning were slightly more persistent during the 8-week follow-up period. Additionally, the participants who more frequently used their anchor during the intervention showed the most persistent meditation routines during the follow-up period, and almost all of these high anchorers were using morning anchors. Our findings suggest that using the anchoring strategy can create persistent morning meditation routines. However, future studies should combine anchoring with additional intervention tools (eg, incentives) to help more participants successfully establish an anchored meditation routine.

## Appendix

Multimedia Appendix 1 CONSORT-eHEALTH checklist (V 1.6.1).

Multimedia Appendix 2 Additional regression results.

## Abbreviations

ANOVA: analysis of variance
CG: control group
FA: fixed anchor
OR: odds ratio
PA: personalized anchor
SRBAI: Self-Report Behavioral Automaticity Index
