# Supplementary material for: Using Personalized Anchors to Establish Routine Meditation Practice With a Mobile App: Randomized Controlled Trial
Source: JMIR Mhealth Uhealth. 2021 Dec 22;9(12):e32794. doi: 10.2196/32794 (PMC8734923; doi:10.2196/32794)
Supplement: Multimedia Appendix 1 [file mhealth_v9i12e32794_app1.pdf]

CONSORT-EHEALTH (V 1.6.1) - Submission/Publication Form

The CONSORT-EHEALTH checklist is intended for authors of randomized trials evaluating web-based and Internet-based applications/interventions, including mobile interventions, electronic games (incl multiplayer games), social media, certain telehealth applications, and other interactive and/or networked electronic applications. Some of the items (e.g. all subitems under item 5 - description of the intervention) may also be applicable for other study designs.

The goal of the CONSORT EHEALTH checklist and guideline is to be
a) a guide for reporting for authors of RCTs,
b) to form a basis for appraisal of an ehealth trial (in terms of validity)

CONSORT-EHEALTH items/subitems are MANDATORY reporting items for studies published in the Journal of Medical Internet Research and other journals / scientific societies endorsing the checklist.

Items numbered 1., 2., 3., 4a., 4b etc are original CONSORT or CONSORT-NPT (non-pharmacologic treatment) items.
Items with Roman numerals (i., ii, iii, iv etc.) are CONSORT-EHEALTH extensions/clarifications.

As the CONSORT-EHEALTH checklist is still considered in a formative stage, we would ask that you also RATE ON A SCALE OF 1-5 how important/useful you feel each item is FOR THE PURPOSE OF THE CHECKLIST and reporting guideline (optional).

Mandatory reporting items are marked with a red *.
In the textboxes, either copy & paste the relevant sections from your manuscript into this form - please include any quotes from your manuscript in QUOTATION MARKS,
or answer directly by providing additional information not in the manuscript, or elaborating on why the item was not relevant for this study.

YOUR ANSWERS WILL BE PUBLISHED AS A SUPPLEMENTARY FILE TO YOUR PUBLICATION IN JMIR AND ARE CONSIDERED PART OF YOUR PUBLICATION (IF ACCEPTED).
Please fill in these questions diligently. Information will not be copyedited, so please use proper spelling and grammar, use correct capitalization, and avoid abbreviations.

DO NOT FORGET TO SAVE AS PDF _AND_ CLICK THE SUBMIT BUTTON SO YOUR ANSWERS

ARE IN OUR DATABASE !!!

Citation Suggestion (if you append the pdf as Appendix we suggest to cite this paper in the caption):
Eysenbach G, CONSORT-EHEALTH Group
CONSORT-EHEALTH: Improving and Standardizing Evaluation Reports of Web-based and Mobile Health Interventions
J Med Internet Res 2011;13(4):e126
URL: http://www.jmir.org/2011/4/e126/
doi: 10.2196/jmir.1923
PMID: 22209829

msulli27@asu.edu (not shared) Switch account Draft not saved

* Required

Your name *

First Last

Chad Stecher

Primary Affiliation (short), City, Country *

University of Toronto, Toronto, Canada

Arizona State University, Phoenix, AZ

Your e-mail address *

abc@gmail.com

chad.stecher@asu.edu

Title of your manuscript *

Provide the (draft) title of your manuscript.

Using Personalized Anchors to Establish Routine Meditation Practice With a Mobile App: Randomized Controlled Trial

Name of your App/Software/Intervention *

If there is a short and a long/alternate name, write the short name first and add the long name in brackets.

Calm [Calm Inc.]

Evaluated Version (if any)

e.g. "V1", "Release 2017-03-01", "Version 2.0.27913"

Your answer

Language(s) *

What language is the intervention/app in? If multiple languages are available, separate by comma (e.g. "English, French")

English

URL of your Intervention Website or App

e.g. a direct link to the mobile app on app in appstore (itunes, Google Play), or URL of the website. If the intervention is a DVD or hardware, you can also link to an Amazon page.

www.Calm.com

URL of an image/screenshot (optional)

Your answer

Accessibility *

Can an enduser access the intervention presently?

- ○ access is free and open
- ○ access only for special usergroups, not open
- ◉ access is open to everyone, but requires payment/subscription/in-app purchases
- ○ app/intervention no longer accessible
- ○ Other:

Primary Medical Indication/Disease/Condition *

e.g. "Stress", "Diabetes", or define the target group in brackets after the condition, e.g. "Autism (Parents of children with)", "Alzheimers (Informal Caregivers of)"

Stress

Primary Outcomes measured in trial *

comma-separated list of primary outcomes reported in the trial

Calm app adherence

Secondary/other outcomes

Are there any other outcomes the intervention is expected to affect?

Your answer

Recommended "Dose" *

What do the instructions for users say on how often the app should be used?

- (x) Approximately Daily
- ( ) Approximately Weekly
- ( ) Approximately Monthly
- ( ) Approximately Yearly
- ( ) "as needed"
- ( ) Other:

Approx. Percentage of Users (starters) still using the app as recommended after 3 months *

- (selected) unknown / not evaluated
- 0-10%
- 11-20%
- 21-30%
- 31-40%
- 41-50%
- 51-60%
- 61-70%
- 71%-80%
- 81-90%
- 91-100%
- Other:

Overall, was the app/intervention effective? *

- ○ yes: all primary outcomes were significantly better in intervention group vs control
- ◉ partly: SOME primary outcomes were significantly better in intervention group vs control
- ○ no statistically significant difference between control and intervention
- ○ potentially harmful: control was significantly better than intervention in one or more outcomes
- ○ inconclusive: more research is needed
- ○ Other:

Article Preparation Status/Stage *

At which stage in your article preparation are you currently (at the time you fill in this form)

- ○ not submitted yet - in early draft status
- ○ not submitted yet - in late draft status, just before submission
- ○ submitted to a journal but not reviewed yet
- ○ submitted to a journal and after receiving initial reviewer comments
- ◉ submitted to a journal and accepted, but not published yet
- ○ published
- ○ Other:

Journal *

If you already know where you will submit this paper (or if it is already submitted), please provide the journal name (if it is not JMIR, provide the journal name under "other")

- ( ) not submitted yet / unclear where I will submit this
- ( ) Journal of Medical Internet Research (JMIR)
- (x) JMIR mHealth and UHealth
- ( ) JMIR Serious Games
- ( ) JMIR Mental Health
- ( ) JMIR Public Health
- ( ) JMIR Formative Research
- ( ) Other JMIR sister journal
- ( ) Other:

Is this a full powered effectiveness trial or a pilot/feasibility trial? *

- (x) Pilot/feasibility
- ( ) Fully powered

Manuscript tracking number *

If this is a JMIR submission, please provide the manuscript tracking number under "other" (The ms tracking number can be found in the submission acknowledgement email, or when you login as author in JMIR. If the paper is already published in JMIR, then the ms tracking number is the four-digit number at the end of the DOI, to be found at the bottom of each published article in JMIR)

- ○ no ms number (yet) / not (yet) submitted to / published in JMIR
- ◉ Other: JMU ms#32794

TITLE AND ABSTRACT

1a) TITLE: Identification as a randomized trial in the title

1a) Does your paper address CONSORT item 1a? *

I.e does the title contain the phrase "Randomized Controlled Trial"? (if not, explain the reason under "other")

- ◉ yes
- ○ Other:

1a-i) Identify the mode of delivery in the title

Identify the mode of delivery. Preferably use “web-based” and/or “mobile” and/or “electronic game” in the title. Avoid ambiguous terms like “online”, “virtual”, “interactive”. Use “Internet-based” only if Intervention includes non-web-based Internet components (e.g. email), use “computer-based” or “electronic” only if offline products are used. Use “virtual” only in the context of “virtual reality” (3-D worlds). Use “online” only in the context of “online support groups”. Complement or substitute product names with broader terms for the class of products (such as “mobile” or “smart phone” instead of “iphone”), especially if the application runs on different platforms.

	1	2	3	4	5	
subitem not at all important	○	○	○	○	◉	essential

Clear selection

Does your paper address subitem 1a-i? *

Copy and paste relevant sections from manuscript title (include quotes in quotation marks "like this" to indicate direct quotes from your manuscript), or elaborate on this item by providing additional information not in the ms, or briefly explain why the item is not applicable/relevant for your study

The title includes the phrase "With a Mobile App"

1a-ii) Non-web-based components or important co-interventions in title

Mention non-web-based components or important co-interventions in title, if any (e.g., “with telephone support”).

	1	2	3	4	5	
subitem not at all important	○	○	○	○	◉	essential

Clear selection

Does your paper address subitem 1a-ii?

Copy and paste relevant sections from manuscript title (include quotes in quotation marks "like this" to indicate direct quotes from your manuscript), or elaborate on this item by providing additional information not in the ms, or briefly explain why the item is not applicable/relevant for your study

This study did not include co-interventions.

1a-iii) Primary condition or target group in the title

Mention primary condition or target group in the title, if any (e.g., "for children with Type I Diabetes")
Example: A Web-based and Mobile Intervention with Telephone Support for Children with Type I Diabetes: Randomized Controlled Trial

	1	2	3	4	5	
subitem not at all important	○	○	○	○	◉	essential

Clear selection

Does your paper address subitem 1a-iii? *

Copy and paste relevant sections from manuscript title (include quotes in quotation marks "like this" to indicate direct quotes from your manuscript), or elaborate on this item by providing additional information not in the ms, or briefly explain why the item is not applicable/relevant for your study

This study did not have a primary condition or target group.

1b) ABSTRACT: Structured summary of trial design, methods, results, and conclusions

NPT extension: Description of experimental treatment, comparator, care providers, centers, and blinding status.

1b-i) Key features/functionalities/components of the intervention and comparator in the METHODS section of the ABSTRACT

Mention key features/functionalities/components of the intervention and comparator in the abstract. If possible, also mention theories and principles used for designing the site. Keep in mind the needs of systematic reviewers and indexers by including important synonyms. (Note: Only report in the abstract what the main paper is reporting. If this information is missing from the main body of text, consider adding it)

	1	2	3	4	5	
subitem not at all important	○	○	○	○	◉	essential

Clear selection

Does your paper address subitem 1b-i? *

Copy and paste relevant sections from the manuscript abstract (include quotes in quotation marks "like this" to indicate direct quotes from your manuscript), or elaborate on this item by providing additional information not in the ms, or briefly explain why the item is not applicable/relevant for your study

We conducted a randomized controlled trial and randomly assigned participants to one of 3 study groups: (1) personalized anchor (PA), (2) fixed anchor (FA), or a (3) control group that did not use the anchoring strategy. All participants received app-delivered reminder messages to meditate for at least 10 minutes a day using the Calm app for an 8-week intervention period, and app usage data continued to be collected for an additional 8-week follow-up period to measure meditation persistence.

1b-ii) Level of human involvement in the METHODS section of the ABSTRACT

Clarify the level of human involvement in the abstract, e.g., use phrases like “fully automated” vs. “therapist/nurse/care provider/physician-assisted” (mention number and expertise of providers involved, if any). (Note: Only report in the abstract what the main paper is reporting. If this information is missing from the main body of text, consider adding it)

	1	2	3	4	5	
subitem not at all important	○	○	○	○	◉	essential

Clear selection

Does your paper address subitem 1b-ii?

Copy and paste relevant sections from the manuscript abstract (include quotes in quotation marks "like this" to indicate direct quotes from your manuscript), or elaborate on this item by providing additional information not in the ms, or briefly explain why the item is not applicable/relevant for your study

The content was app-delivered: "All participants received app-delivered reminder messages to meditate for at least 10 minutes a day using the Calm app for an 8-week intervention period".

1b-iii) Open vs. closed, web-based (self-assessment) vs. face-to-face assessments in the METHODS section of the ABSTRACT

Mention how participants were recruited (online vs. offline), e.g., from an open access website or from a clinic or a closed online user group (closed usergroup trial), and clarify if this was a purely web-based trial, or there were face-to-face components (as part of the intervention or for assessment). Clearly say if outcomes were self-assessed through questionnaires (as common in web-based trials). Note: In traditional offline trials, an open trial (open-label trial) is a type of clinical trial in which both the researchers and participants know which treatment is being administered. To avoid confusion, use “blinded” or “unblinded” to indicated the level of blinding instead of “open”, as “open” in web-based trials usually refers to “open access” (i.e. participants can self-enrol). (Note: Only report in the abstract what the main paper is reporting. If this information is missing from the main body of text, consider adding it)

	1	2	3	4	5	
subitem not at all important	○	○	○	○	◉	essential

Clear selection

Does your paper address subitem 1b-iii?

Copy and paste relevant sections from the manuscript abstract (include quotes in quotation marks "like this" to indicate direct quotes from your manuscript), or elaborate on this item by providing additional information not in the ms, or briefly explain why the item is not applicable/relevant for your study

Participants were recruited online. This trial was purely web-based. Outcomes were assessed via questionnaires: "Baseline, week 8, and week 16 surveys were administered to assess demographics, socioeconomic status, and changes in self-reported habit strength."

1b-iv) RESULTS section in abstract must contain use data

Report number of participants enrolled/assessed in each group, the use/uptake of the intervention (e.g., attrition/adherence metrics, use over time, number of logins etc.), in addition to primary/secondary outcomes. (Note: Only report in the abstract what the main paper is reporting. If this information is missing from the main body of text, consider adding it)

	1	2	3	4	5	
subitem not at all important	○	○	○	○	◉	essential

Clear selection

Does your paper address subitem 1b-iv?

Copy and paste relevant sections from the manuscript abstract (include quotes in quotation marks "like this" to indicate direct quotes from your manuscript), or elaborate on this item by providing additional information not in the ms, or briefly explain why the item is not applicable/relevant for your study

"A total of 101 participants across the 3 study groups were included in the final analysis: (1) PA (n=56), (2) FA (n=49), and (3) control group (n=62)."
"The FA group had a significantly higher average odds of daily meditation during the intervention (1.14 odds ratio [OR]; 95% CI 1.02-1.33; P=.04), and all participants experienced a linear decline in their odds of daily meditation during the 8-week intervention (0.96 OR; 95% CI 0.95-0.96; P<.001). Importantly, the FA group showed a significantly smaller decline in the linear trend of their odds of daily meditation during the 8-week follow-up (their daily trend increased by 1.04 OR from their trend during the intervention; 95% CI 1.01-1.06; P=.03). Additionally, those who more frequently adhered to their anchoring strategy during the intervention typically used anchors that occurred in the morning and showed a significantly smaller decline in their odds of daily meditation during the 8-week follow-up period (1.13 OR; 95% CI 1.02-1.35; P=.007)."

1b-v) CONCLUSIONS/DISCUSSION in abstract for negative trials

Conclusions/Discussions in abstract for negative trials: Discuss the primary outcome - if the trial is negative (primary outcome not changed), and the intervention was not used, discuss whether negative results are attributable to lack of uptake and discuss reasons. (Note: Only report in the abstract what the main paper is reporting. If this information is missing from the main body of text, consider adding it)

	1	2	3	4	5	
subitem not at all important	○	○	○	○	◉	essential

Clear selection

Does your paper address subitem 1b-v?

Copy and paste relevant sections from the manuscript abstract (include quotes in quotation marks "like this" to indicate direct quotes from your manuscript), or elaborate on this item by providing additional information not in the ms, or briefly explain why the item is not applicable/relevant for your study

"The FA group had more persistent meditation with the app, but participants in the FA or PA groups who more frequently adhered to their anchoring strategy during the intervention had the most persistent meditation routines, and almost all of these high anchorers used morning anchors. These findings suggest that the anchoring strategy can create persistent meditation routines with a mobile app. However, future studies should combine anchoring with additional intervention tools (eg, incentives) to help more participants successfully establish an anchored meditation routine."

INTRODUCTION

2a) In INTRODUCTION: Scientific background and explanation of rationale

2a-i) Problem and the type of system/solution

Describe the problem and the type of system/solution that is object of the study: intended as stand-alone intervention vs. incorporated in broader health care program? Intended for a particular patient population? Goals of the intervention, e.g., being more cost-effective to other interventions, replace or complement other solutions? (Note: Details about the intervention are provided in “Methods” under 5)

	1	2	3	4	5	
subitem not at all important	○	○	○	○	○	essential

Does your paper address subitem 2a-i? *

Copy and paste relevant sections from the manuscript (include quotes in quotation marks "like this" to indicate direct quotes from your manuscript), or elaborate on this item by providing additional information not in the ms, or briefly explain why the item is not applicable/relevant for your study

The goal of this research was to determine how to best promote adherence to meditation app use: "the purpose of this study was to test the efficacy of using a personalized anchor versus having an anchor assigned in the morning (ie, fixed) for successfully establishing a persistent meditation app routine using the mobile app Calm."

2a-ii) Scientific background, rationale: What is known about the (type of) system

Scientific background, rationale: What is known about the (type of) system that is the object of the study (be sure to discuss the use of similar systems for other conditions/diagnoses, if appropiate), motivation for the study, i.e. what are the reasons for and what is the context for this specific study, from which stakeholder viewpoint is the study performed, potential impact of findings [2]. Briefly justify the choice of the comparator.

	1	2	3	4	5	
subitem not at all important	○	○	○	○	○	essential

Does your paper address subitem 2a-ii? *

Copy and paste relevant sections from the manuscript (include quotes in quotation marks "like this" to indicate direct quotes from your manuscript), or elaborate on this item by providing additional information not in the ms, or briefly explain why the item is not applicable/relevant for your study

"Psychology research has shown that behaviors consistently performed in response to the same contextual (or environmental) cue become routinized, meaning they are completed with little or no cognitive effort... One successful strategy for establishing a new routine is anchoring or pairing the new behavior to an existing routine that is already executed with very little cognitive effort."

2b) In INTRODUCTION: Specific objectives or hypotheses

Does your paper address CONSORT subitem 2b? *

Copy and paste relevant sections from the manuscript (include quotes in quotation marks "like this" to indicate direct quotes from your manuscript), or elaborate on this item by providing additional information not in the ms, or briefly explain why the item is not applicable/relevant for your study

"We hypothesized that the personalized anchor group would be the most persistent over the 8-week follow-up period and that both intervention groups would have significantly greater meditation persistence relative to the control group."

METHODS

3a) Description of trial design (such as parallel, factorial) including allocation ratio

Does your paper address CONSORT subitem 3a? *

Copy and paste relevant sections from the manuscript (include quotes in quotation marks "like this" to indicate direct quotes from your manuscript), or elaborate on this item by providing additional information not in the ms, or briefly explain why the item is not applicable/relevant for your study

"A randomized controlled trial was conducted between July 2020 and March 2021 with an 8-week intervention period, an 8-week follow-up period, and survey assessments at baseline, week 8, and week 16. The Institutional Review Board at Arizona State University approved this study (STUDY00011788), and all participants provided consent electronically prior to participating in the survey.
Recruitment
Study recruitment took place from July to August 2020. Participants were paying subscribers to the Calm app who were identified as not having already formed a daily meditation routine. Specifically, subscribers were eligible if they had subscribed to the Calm app after January 2020, had not completed a meditation session with the app in the past 30 days, and did not report practicing meditation with or without the app for more than 60 minutes in 1 month over the past 6 months. Additionally, new subscribers were eligible if they could read and understand English, were willing to be randomized, and were between 18 and 60 years old (see Textbox 1 for a full list of study eligibility criteria). Eligible subscribers were identified by Calm and invited to participate in the study via email. The email contained a brief overview of the study and a link to a short eligibility survey using Qualtrics software to verify that participants satisfied all remaining study eligibility criteria. Eligible participants were then automatically directed to read and electronically sign an informed consent document in Qualtrics. Consenting participants were then contacted by the research team via email to complete the baseline questionnaire in Qualtrics. Once complete, participants were randomized to 1 of 3 study groups using a predetermined allocation list generated on Randomizer.org by a researcher not involved in the participant assignment. Participants were then assigned to a study group based on the allocation list and the order in which they were enrolled in the study."

3b) Important changes to methods after trial commencement (such as eligibility criteria), with reasons

Does your paper address CONSORT subitem 3b? *

Copy and paste relevant sections from the manuscript (include quotes in quotation marks "like this" to indicate direct quotes from your manuscript), or elaborate on this item by providing additional information not in the ms, or briefly explain why the item is not applicable/relevant for your study

There were no changes after trial commencement.

3b-i) Bug fixes, Downtimes, Content Changes

Bug fixes, Downtimes, Content Changes: ehealth systems are often dynamic systems. A description of changes to methods therefore also includes important changes made on the intervention or comparator during the trial (e.g., major bug fixes or changes in the functionality or content) (5-iii) and other “unexpected events” that may have influenced study design such as staff changes, system failures/downtimes, etc. [2].

	1	2	3	4	5	
subitem not at all important	○	○	○	○	○	essential

Does your paper address subitem 3b-i?

Copy and paste relevant sections from the manuscript (include quotes in quotation marks "like this" to indicate direct quotes from your manuscript), or elaborate on this item by providing additional information not in the ms, or briefly explain why the item is not applicable/relevant for your study

Your answer

4a) Eligibility criteria for participants

Does your paper address CONSORT subitem 4a? *

Copy and paste relevant sections from the manuscript (include quotes in quotation marks "like this" to indicate direct quotes from your manuscript), or elaborate on this item by providing additional information not in the ms, or briefly explain why the item is not applicable/relevant for your study

"Inclusion criteria
• 18-60 years of age
• Purchased Calm after January 2020
• Inactive: have not used app in the past 30 days
• Own an iOS/Android smartphone
• Own home internet or unlimited data plan
• Able to read and understand English
• Willing to be randomized

Exclusion criteria
• Report practicing mindfulness meditation
>60 min in 1 month within the last 6 months
• Any meditation sessions with app in the past last 30 days
• Currently reside outside the USA"

4a-i) Computer / Internet literacy

Computer / Internet literacy is often an implicit “de facto” eligibility criterion - this should be explicitly clarified.

	1	2	3	4	5	
subitem not at all important	○	○	○	○	○	essential

Does your paper address subitem 4a-i?

Copy and paste relevant sections from the manuscript (include quotes in quotation marks "like this" to indicate direct quotes from your manuscript), or elaborate on this item by providing additional information not in the ms, or briefly explain why the item is not applicable/relevant for your study

Your answer

4a-ii) Open vs. closed, web-based vs. face-to-face assessments:

Open vs. closed, web-based vs. face-to-face assessments: Mention how participants were recruited (online vs. offline), e.g., from an open access website or from a clinic, and clarify if this was a purely web-based trial, or there were face-to-face components (as part of the intervention or for assessment), i.e., to what degree got the study team to know the participant. In online-only trials, clarify if participants were quasi-anonymous and whether having multiple identities was possible or whether technical or logistical measures (e.g., cookies, email confirmation, phone calls) were used to detect/prevent these.

	1	2	3	4	5	
subitem not at all important	○	○	○	○	○	essential

Does your paper address subitem 4a-ii? *

Copy and paste relevant sections from the manuscript (include quotes in quotation marks "like this" to indicate direct quotes from your manuscript), or elaborate on this item by providing additional information not in the ms, or briefly explain why the item is not applicable/relevant for your study

"Eligible subscribers were identified by Calm and invited to participate in the study via email. The email contained a brief overview of the study and a link to a short eligibility survey using Qualtrics software to verify that participants satisfied all remaining study eligibility criteria. Eligible participants were then automatically directed to read and electronically sign an informed consent document in Qualtrics. Consenting participants were then contacted by the research team via email to complete the baseline questionnaire in Qualtrics. "

4a-iii) Information giving during recruitment

Information given during recruitment. Specify how participants were briefed for recruitment and in the informed consent procedures (e.g., publish the informed consent documentation as appendix, see also item X26), as this information may have an effect on user self-selection, user expectation and may also bias results.

	1	2	3	4	5	
subitem not at all important	○	○	○	○	○	essential

Does your paper address subitem 4a-iii?

Copy and paste relevant sections from the manuscript (include quotes in quotation marks "like this" to indicate direct quotes from your manuscript), or elaborate on this item by providing additional information not in the ms, or briefly explain why the item is not applicable/relevant for your study

Your answer

4b) Settings and locations where the data were collected

Does your paper address CONSORT subitem 4b? *

Copy and paste relevant sections from the manuscript (include quotes in quotation marks "like this" to indicate direct quotes from your manuscript), or elaborate on this item by providing additional information not in the ms, or briefly explain why the item is not applicable/relevant for your study

"Eligible subscribers were identified by Calm and invited to participate in the study via email. The email contained a brief overview of the study and a link to a short eligibility survey using Qualtrics software to verify that participants satisfied all remaining study eligibility criteria. Eligible participants were then automatically directed to read and electronically sign an informed consent document in Qualtrics. Consenting participants were then contacted by the research team via email to complete the baseline questionnaire in Qualtrics. "

4b-i) Report if outcomes were (self-)assessed through online questionnaires

Clearly report if outcomes were (self-)assessed through online questionnaires (as common in web-based trials) or otherwise.

	1	2	3	4	5	
subitem not at all important	○	○	○	○	○	essential

Does your paper address subitem 4b-i? *

Copy and paste relevant sections from the manuscript (include quotes in quotation marks "like this" to indicate direct quotes from your manuscript), or elaborate on this item by providing additional information not in the ms, or briefly explain why the item is not applicable/relevant for your study

"The baseline, postintervention, and final questionnaires were all completed in Qualtrics. Participants were asked to respond using “A little bit,” “Neutral,” “Quite a bit,” or “A lot” to the following 3 questions about the COVID-19 pandemic: “To what extent do you feel the COVID-19 pandemic has affected your mental health?”, “To what extent do you feel the COVID-19 pandemic has affected your physical health?”, and “To what extent do you feel the COVID-19 pandemic has affected your stress?” Participants also completed the Self-Report Behavioral Automaticity Index (SRBAI) on each survey to assess the strength of their meditation habit (ie, self-reported habit strength) [54]. The SRBAI contains 4 items scored on a 5-point Likert scale from 1 “Strongly disagree” to 5 “Strongly agree” in response to statements like “Daily meditation is something I do automatically,” where a higher sum of item scores indicates a stronger habit. The SRBAI has a Cronbach’s α of ≥.81 and was designed using discriminant content validity while preserving strong predictive validity [54]. Each survey also asked participants to rate their overall health as either “Poor,” “Fair,” “Good,” “Very Good,” or “Excellent.” On the baseline survey, participants answered questions on their demographic and socioeconomic characteristics. "

4b-ii) Report how institutional affiliations are displayed

Report how institutional affiliations are displayed to potential participants [on ehealth media], as affiliations with prestigious hospitals or universities may affect volunteer rates, use, and reactions with regards to an intervention.(Not a required item – describe only if this may bias results)

	1	2	3	4	5	
subitem not at all important	○	○	○	○	○	essential

Does your paper address subitem 4b-ii?

Copy and paste relevant sections from the manuscript (include quotes in quotation marks "like this" to indicate direct quotes from your manuscript), or elaborate on this item by providing additional information not in the ms, or briefly explain why the item is not applicable/relevant for your study

Your answer

5) The interventions for each group with sufficient details to allow replication, including how and when they were actually administered

5-i) Mention names, credential, affiliations of the developers, sponsors, and owners

Mention names, credential, affiliations of the developers, sponsors, and owners [6] (if authors/evaluators are owners or developer of the software, this needs to be declared in a “Conflict of interest” section or mentioned elsewhere in the manuscript).

	1	2	3	4	5	
subitem not at all important	○	○	○	○	○	essential

Does your paper address subitem 5-i?

Copy and paste relevant sections from the manuscript (include quotes in quotation marks "like this" to indicate direct quotes from your manuscript), or elaborate on this item by providing additional information not in the ms, or briefly explain why the item is not applicable/relevant for your study

Your answer

5-ii) Describe the history/development process

Describe the history/development process of the application and previous formative evaluations (e.g., focus groups, usability testing), as these will have an impact on adoption/use rates and help with interpreting results.

	1	2	3	4	5	
subitem not at all important	○	○	○	○	○	essential

Does your paper address subitem 5-ii?

Copy and paste relevant sections from the manuscript (include quotes in quotation marks "like this" to indicate direct quotes from your manuscript), or elaborate on this item by providing additional information not in the ms, or briefly explain why the item is not applicable/relevant for your study

Your answer

5-iii) Revisions and updating

Revisions and updating. Clearly mention the date and/or version number of the application/intervention (and comparator, if applicable) evaluated, or describe whether the intervention underwent major changes during the evaluation process, or whether the development and/or content was “frozen” during the trial. Describe dynamic components such as news feeds or changing content which may have an impact on the replicability of the intervention (for unexpected events see item 3b).

	1	2	3	4	5	
subitem not at all important	○	○	○	○	○	essential

Does your paper address subitem 5-iii?

Copy and paste relevant sections from the manuscript (include quotes in quotation marks "like this" to indicate direct quotes from your manuscript), or elaborate on this item by providing additional information not in the ms, or briefly explain why the item is not applicable/relevant for your study

Your answer

5-iv) Quality assurance methods

Provide information on quality assurance methods to ensure accuracy and quality of information provided [1], if applicable.

	1	2	3	4	5	
subitem not at all important	○	○	○	○	○	essential

Does your paper address subitem 5-iv?

Copy and paste relevant sections from the manuscript (include quotes in quotation marks "like this" to indicate direct quotes from your manuscript), or elaborate on this item by providing additional information not in the ms, or briefly explain why the item is not applicable/relevant for your study

Your answer

5-v) Ensure replicability by publishing the source code, and/or providing screenshots/screen-capture video, and/or providing flowcharts of the algorithms used

Ensure replicability by publishing the source code, and/or providing screenshots/screen-capture video, and/or providing flowcharts of the algorithms used. Replicability (i.e., other researchers should in principle be able to replicate the study) is a hallmark of scientific reporting.

	1	2	3	4	5	
subitem not at all important	○	○	○	○	○	essential

Does your paper address subitem 5-v?

Copy and paste relevant sections from the manuscript (include quotes in quotation marks "like this" to indicate direct quotes from your manuscript), or elaborate on this item by providing additional information not in the ms, or briefly explain why the item is not applicable/relevant for your study

Your answer

5-vi) Digital preservation

Digital preservation: Provide the URL of the application, but as the intervention is likely to change or disappear over the course of the years; also make sure the intervention is archived (Internet Archive, webcitation.org, and/or publishing the source code or screenshots/videos alongside the article). As pages behind login screens cannot be archived, consider creating demo pages which are accessible without login.

	1	2	3	4	5	
subitem not at all important	○	○	○	○	○	essential

Does your paper address subitem 5-vi?

Copy and paste relevant sections from the manuscript (include quotes in quotation marks "like this" to indicate direct quotes from your manuscript), or elaborate on this item by providing additional information not in the ms, or briefly explain why the item is not applicable/relevant for your study

Your answer

5-vii) Access

Access: Describe how participants accessed the application, in what setting/context, if they had to pay (or were paid) or not, whether they had to be a member of specific group. If known, describe how participants obtained “access to the platform and Internet” [1]. To ensure access for editors/reviewers/readers, consider to provide a “backdoor” login account or demo mode for reviewers/readers to explore the application (also important for archiving purposes, see vi).

	1	2	3	4	5	
subitem not at all important	○	○	○	○	○	essential

Does your paper address subitem 5-vii? *

Copy and paste relevant sections from the manuscript (include quotes in quotation marks "like this" to indicate direct quotes from your manuscript), or elaborate on this item by providing additional information not in the ms, or briefly explain why the item is not applicable/relevant for your study

"During the 8-week intervention period, all participants received a daily app–delivered reminder message (ie, push notification) to either meditate for at least 10 minutes or to meditate for at least 10 minutes using their anchor. Messages were randomly delivered at either 8 AM, 1 PM, or 6 PM (ie, a 33.3% chance of receiving the daily message at 1 of the 3 possible times), with adjustments made for participants' time zone. The message content was also randomized with a 50% chance of receiving 1 of 2 message types. The first message type included study group–specific reminders reinforcing participants' use of either their personalized or fixed anchors, or reminding the control group to meditate. The second message type was evenly randomized between reminders to use 3 motivational tools in the Calm app: mood check-ins, the meditation activity tracker, or the in-app daily reminder tool. The success of each type, timing, and sequence of daily supports was evaluated based on both participants' daily app usage data and ecological momentary assessments collected via SMS text message once per evening (8 PM) during the 8-week intervention. The results from this microrandomized trial on the effectiveness of different daily reminder messages are not reported in this paper, and it is important to note that this microrandomized trial study design meant that each message type, timing, and sequence were randomly delivered across all study groups; thus, the sequence of messages would not bias our analysis of the overall study group differences in meditation persistence during this study.
Participants were initially instructed to use their anchors (PA and FA groups) and meditate for 10 minutes per day (all groups) for 8 weeks. After 8 weeks, participants were emailed a postintervention survey to complete and were encouraged to continue meditating but were not given further instructions. Participants were emailed again at the end of the 8-week follow-up period and given a final questionnaire to complete. "

5-viii) Mode of delivery, features/functionalities/components of the intervention and comparator, and the theoretical framework

Describe mode of delivery, features/functionalities/components of the intervention and comparator, and the theoretical framework [6] used to design them (instructional strategy [1], behaviour change techniques, persuasive features, etc., see e.g., [7, 8] for terminology). This includes an in-depth description of the content (including where it is coming from and who developed it) [1],” whether [and how] it is tailored to individual circumstances and allows users to track their progress and receive feedback” [6]. This also includes a description of communication delivery channels and – if computer-mediated communication is a component – whether communication was synchronous or asynchronous [6]. It also includes information on presentation strategies [1], including page design principles, average amount of text on pages, presence of hyperlinks to other resources, etc. [1].

	1	2	3	4	5	
subitem not at all important	○	○	○	○	○	essential

Does your paper address subitem 5-viii? *

Copy and paste relevant sections from the manuscript (include quotes in quotation marks "like this" to indicate direct quotes from your manuscript), or elaborate on this item by providing additional information not in the ms, or briefly explain why the item is not applicable/relevant for your study

"During the 8-week intervention period, all participants received a daily app–delivered reminder message (ie, push notification) to either meditate for at least 10 minutes or to meditate for at least 10 minutes using their anchor. Messages were randomly delivered at either 8 AM, 1 PM, or 6 PM (ie, a 33.3% chance of receiving the daily message at 1 of the 3 possible times), with adjustments made for participants' time zone. The message content was also randomized with a 50% chance of receiving 1 of 2 message types. The first message type included study group–specific reminders reinforcing participants' use of either their personalized or fixed anchors, or reminding the control group to meditate. The second message type was evenly randomized between reminders to use 3 motivational tools in the Calm app: mood check-ins, the meditation activity tracker, or the in-app daily reminder tool. The success of each type, timing, and sequence of daily supports was evaluated based on both participants' daily app usage data and ecological momentary assessments collected via SMS text message once per evening (8 PM) during the 8-week intervention. The results from this microrandomized trial on the effectiveness of different daily reminder messages are not reported in this paper, and it is important to note that this microrandomized trial study design meant that each message type, timing, and sequence were randomly delivered across all study groups; thus, the sequence of messages would not bias our analysis of the overall study group differences in meditation persistence during this study."

5-ix) Describe use parameters

Describe use parameters (e.g., intended "doses" and optimal timing for use). Clarify what instructions or recommendations were given to the user, e.g., regarding timing, frequency, heaviness of use, if any, or was the intervention used ad libitum.

	1	2	3	4	5	
subitem not at all important	○	○	○	○	○	essential

Does your paper address subitem 5-ix?

Copy and paste relevant sections from the manuscript (include quotes in quotation marks "like this" to indicate direct quotes from your manuscript), or elaborate on this item by providing additional information not in the ms, or briefly explain why the item is not applicable/relevant for your study

Your answer

5-x) Clarify the level of human involvement

Clarify the level of human involvement (care providers or health professionals, also technical assistance) in the e-intervention or as co-intervention (detail number and expertise of professionals involved, if any, as well as “type of assistance offered, the timing and frequency of the support, how it is initiated, and the medium by which the assistance is delivered”. It may be necessary to distinguish between the level of human involvement required for the trial, and the level of human involvement required for a routine application outside of a RCT setting (discuss under item 21 – generalizability).

	1	2	3	4	5	
subitem not at all important	○	○	○	○	○	essential

Does your paper address subitem 5-x?

Copy and paste relevant sections from the manuscript (include quotes in quotation marks "like this" to indicate direct quotes from your manuscript), or elaborate on this item by providing additional information not in the ms, or briefly explain why the item is not applicable/relevant for your study

Your answer

5-xi) Report any prompts/reminders used

Report any prompts/reminders used: Clarify if there were prompts (letters, emails, phone calls, SMS) to use the application, what triggered them, frequency etc. It may be necessary to distinguish between the level of prompts/reminders required for the trial, and the level of prompts/reminders for a routine application outside of a RCT setting (discuss under item 21 – generalizability).

	1	2	3	4	5	
subitem not at all important	○	○	○	○	○	essential

Does your paper address subitem 5-xi? *

Copy and paste relevant sections from the manuscript (include quotes in quotation marks "like this" to indicate direct quotes from your manuscript), or elaborate on this item by providing additional information not in the ms, or briefly explain why the item is not applicable/relevant for your study

"During the 8-week intervention period, all participants received a daily app–delivered reminder message (ie, push notification) to either meditate for at least 10 minutes or to meditate for at least 10 minutes using their anchor. Messages were randomly delivered at either 8 AM, 1 PM, or 6 PM (ie, a 33.3% chance of receiving the daily message at 1 of the 3 possible times), with adjustments made for participants' time zone. The message content was also randomized with a 50% chance of receiving 1 of 2 message types. The first message type included study group–specific reminders reinforcing participants' use of either their personalized or fixed anchors, or reminding the control group to meditate. The second message type was evenly randomized between reminders to use 3 motivational tools in the Calm app: mood check-ins, the meditation activity tracker, or the in-app daily reminder tool. The success of each type, timing, and sequence of daily supports was evaluated based on both participants' daily app usage data and ecological momentary assessments collected via SMS text message once per evening (8 PM) during the 8-week intervention. The results from this microrandomized trial on the effectiveness of different daily reminder messages are not reported in this paper, and it is important to note that this microrandomized trial study design meant that each message type, timing, and sequence were randomly delivered across all study groups; thus, the sequence of messages would not bias our analysis of the overall study group differences in meditation persistence during this study.
Participants were initially instructed to use their anchors (PA and FA groups) and meditate for 10 minutes per day (all groups) for 8 weeks. After 8 weeks, participants were emailed a postintervention survey to complete and were encouraged to continue meditating but were not given further instructions. Participants were emailed again at the end of the 8-week follow-up period and given a final questionnaire to complete."

5-xii) Describe any co-interventions (incl. training/support)

Describe any co-interventions (incl. training/support): Clearly state any interventions that are provided in addition to the targeted eHealth intervention, as ehealth intervention may not be designed as stand-alone intervention. This includes training sessions and support [1]. It may be necessary to distinguish between the level of training required for the trial, and the level of training for a routine application outside of a RCT setting (discuss under item 21 – generalizability.

	1	2	3	4	5	
subitem not at all important	○	○	○	○	○	essential

Does your paper address subitem 5-xii? *

Copy and paste relevant sections from the manuscript (include quotes in quotation marks "like this" to indicate direct quotes from your manuscript), or elaborate on this item by providing additional information not in the ms, or briefly explain why the item is not applicable/relevant for your study

"Intervention
Participants were randomized into a personalized anchor (PA) group, fixed anchor (FA) group, or control group (CG). Participants in this study used their own, paid Calm accounts to access the app during the study. After completing the baseline survey, participants were sent a link to watch an instructional video that provided information about the benefits of meditating 10 minutes per day and study group–specific instructions on how to participate in the study. For those in the PA group, the video instructed participants to select an existing routine to which they would anchor their 10 minutes of daily meditation practice. The PA group's instructional video emphasized the importance of selecting a consistently occurring daily routine that could reliably be followed by 10 or more minutes of meditation and provided clear examples of such existing routines (eg, "After I finish my coffee in the afternoon" or "After I finish breakfast in the morning"). For those in the FA group, participants were instructed to use a fixed anchor provided by the research team to which they would anchor their 10 minutes of daily meditation practice. The anchor provided was the following: "After I finish in the bathroom (brushing teeth, removing mouth guard, etc.) in the morning, I will meditate for at least 10 minutes." Participants in the CG were given information about the mental health benefits of meditating for at least 10 minutes per day and instructed to complete 10 minutes of daily meditation but were not given any instruction on how or when to meditate. Participants were blinded to the other intervention protocols and did not know what intervention component was the focus of this study. To verify participants' comprehension of their study group–specific instructions, participants completed a 3-question comprehension quiz in Qualtrics and were given unlimited chances

to answer each question correctly. Once all questions were correctly answered, participants were emailed with a start date for their intervention and they were provided with a written copy of the study instructions.
During the 8-week intervention period, all participants received a daily app–delivered reminder message (ie, push notification) to either meditate for at least 10 minutes or to meditate for at least 10 minutes using their anchor. Messages were randomly delivered at either 8 AM, 1 PM, or 6 PM (ie, a 33.3% chance of receiving the daily message at 1 of the 3 possible times), with adjustments made for participants' time zone. The message content was also randomized with a 50% chance of receiving 1 of 2 message types. The first message type included study group–specific reminders reinforcing participants' use of either their personalized or fixed anchors, or reminding the control group to meditate. The second message type was evenly randomized between reminders to use 3 motivational tools in the Calm app: mood check-ins, the meditation activity tracker, or the in-app daily reminder tool. The success of each type, timing, and sequence of daily supports was evaluated based on both participants' daily app usage data and ecological momentary assessments collected via SMS text message once per evening (8 PM) during the 8-week intervention. The results from this microrandomized trial on the effectiveness of different daily reminder messages are not reported in this paper, and it is important to note that this microrandomized trial study design meant that each message type, timing, and sequence were randomly delivered across all study groups; thus, the sequence of messages would not bias our analysis of the overall study group differences in meditation persistence during this study.
Participants were initially instructed to use their anchors (PA and FA groups) and meditate for 10 minutes per day (all groups) for 8 weeks. After 8 weeks, participants were emailed a postintervention survey to complete and were encouraged to continue meditating but were not given further instructions. Participants were emailed again at the end of the 8-week follow-up period and given a final questionnaire to complete."

6a) Completely defined pre-specified primary and secondary outcome measures, including how and when they were assessed

Does your paper address CONSORT subitem 6a? *

Copy and paste relevant sections from the manuscript (include quotes in quotation marks "like this" to indicate direct quotes from your manuscript), or elaborate on this item by providing additional information not in the ms, or briefly explain why the item is not applicable/relevant for your study

"Surveys
The baseline, postintervention, and final questionnaires were all completed in Qualtrics. Participants were asked to respond using “A little bit,” “Neutral,” “Quite a bit,” or “A lot” to the following 3 questions about the COVID-19 pandemic: “To what extent do you feel the COVID-19 pandemic has affected your mental health?”, “To what extent do you feel the COVID-19 pandemic has affected your physical health?”, and “To what extent do you feel the COVID-19 pandemic has affected your stress?” Participants also completed the Self-Report Behavioral Automaticity Index (SRBAI) on each survey to assess the strength of their meditation habit (ie, self-reported habit strength) [54]. The SRBAI contains 4 items scored on a 5-point Likert scale from 1 “Strongly disagree” to 5 “Strongly agree” in response to statements like “Daily meditation is something I do automatically,” where a higher sum of item scores indicates a stronger habit. The SRBAI has a Cronbach’s α of ≥.81 and was designed using discriminant content validity while preserving strong predictive validity [54]. Each survey also asked participants to rate their overall health as either “Poor,” “Fair,” “Good,” “Very Good,” or “Excellent.” On the baseline survey, participants answered questions on their demographic and socioeconomic characteristics.
Outcomes
The primary outcome measure for this study was a binary measure of any daily meditation over the 16-week study, which was derived from participants’ Calm app usage data provided by the Calm analytics team. Specifically, we used minute-level data on the time of day and duration of meditation sessions with the Calm app to construct an indicator variable equal to one if a participant completed any minutes of meditation on a given day, and zero otherwise. To study how our intervention impacted meditation persistence, we examine how the odds of performing any daily meditation changed over time both during and after the intervention. The app usage data were also used to construct an indicator variable equal to one if a participant completed any minutes of meditation within 1 hour of the typical time that their personalized anchor was reported to occur (this typical time was collected when the PA group selected their anchor) or when the fixed anchor was expected to occur (8 AM). This measure of temporally consistent meditation was used to study participants’ adherence to their anchoring strategy during and after the intervention. The secondary outcome of interest was the change in SRBAI between the study groups. "

6a-i) Online questionnaires: describe if they were validated for online use and apply CHERRIES items to describe how the questionnaires were designed/deployed

If outcomes were obtained through online questionnaires, describe if they were validated for online use and apply CHERRIES items to describe how the questionnaires were designed/deployed [9].

	1	2	3	4	5	
subitem not at all important	○	○	○	○	○	essential

Does your paper address subitem 6a-i?

Copy and paste relevant sections from manuscript text

Your answer

6a-ii) Describe whether and how “use” (including intensity of use/dosage) was defined/measured/monitored

Describe whether and how “use” (including intensity of use/dosage) was defined/measured/monitored (logins, logfile analysis, etc.). Use/adoption metrics are important process outcomes that should be reported in any ehealth trial.

	1	2	3	4	5	
subitem not at all important	○	○	○	○	○	essential

Does your paper address subitem 6a-ii?

Copy and paste relevant sections from manuscript text

Your answer

6a-iii) Describe whether, how, and when qualitative feedback from participants was obtained

Describe whether, how, and when qualitative feedback from participants was obtained (e.g., through emails, feedback forms, interviews, focus groups).

	1	2	3	4	5	
subitem not at all important	○	○	○	○	○	essential

Does your paper address subitem 6a-iii?

Copy and paste relevant sections from manuscript text

Your answer

6b) Any changes to trial outcomes after the trial commenced, with reasons

Does your paper address CONSORT subitem 6b? *

Copy and paste relevant sections from the manuscript (include quotes in quotation marks "like this" to indicate direct quotes from your manuscript), or elaborate on this item by providing additional information not in the ms, or briefly explain why the item is not applicable/relevant for your study

There were no changes to the trial outcomes.

7a) How sample size was determined

NPT: When applicable, details of whether and how the clustering by care provides or centers was addressed

7a-i) Describe whether and how expected attrition was taken into account when calculating the sample size

Describe whether and how expected attrition was taken into account when calculating the sample size.

	1	2	3	4	5	
subitem not at all important	○	○	○	○	○	essential

Does your paper address subitem 7a-i?

Copy and paste relevant sections from manuscript title (include quotes in quotation marks "like this" to indicate direct quotes from your manuscript), or elaborate on this item by providing additional information not in the ms, or briefly explain why the item is not applicable/relevant for your study

"A total sample size of 150 participants (study group sizes of 50) was targeted based on our available resources, and our expected statistical power was informed by prior interventions using the Calm app [3,55,56]. Assuming a small- to medium-effect size of 0.20, study group sizes of 50 yielded a statistical power of $1-\beta = .76$ for detecting study group x day–level differences in linear models of our repeated daily outcome (any meditation minutes) over the 16-week study at $\alpha = .05$ (calculated using GLIMMPSE [57])."

7b) When applicable, explanation of any interim analyses and stopping guidelines

Does your paper address CONSORT subitem 7b? *

Copy and paste relevant sections from the manuscript (include quotes in quotation marks "like this" to indicate direct quotes from your manuscript), or elaborate on this item by providing additional information not in the ms, or briefly explain why the item is not applicable/relevant for your study

This question is not applicable.

8a) Method used to generate the random allocation sequence

NPT: When applicable, how care providers were allocated to each trial group

Does your paper address CONSORT subitem 8a? *

Copy and paste relevant sections from the manuscript (include quotes in quotation marks "like this" to indicate direct quotes from your manuscript), or elaborate on this item by providing additional information not in the ms, or briefly explain why the item is not applicable/relevant for your study

"Once complete, participants were randomized to 1 of 3 study groups using a predetermined allocation list generated on Randomizer.org by a researcher not involved in the participant assignment. Participants were then assigned to a study group based on the allocation list and the order in which they were enrolled in the study. "

8b) Type of randomisation; details of any restriction (such as blocking and block size)

Does your paper address CONSORT subitem 8b? *

Copy and paste relevant sections from the manuscript (include quotes in quotation marks "like this" to indicate direct quotes from your manuscript), or elaborate on this item by providing additional information not in the ms, or briefly explain why the item is not applicable/relevant for your study

"Once complete, participants were randomized to 1 of 3 study groups using a predetermined allocation list generated on Randomizer.org by a researcher not involved in the participant assignment. Participants were then assigned to a study group based on the allocation list and the order in which they were enrolled in the study. "

9) Mechanism used to implement the random allocation sequence (such as sequentially numbered containers), describing any steps taken to conceal the sequence until interventions were assigned

Does your paper address CONSORT subitem 9? *

Copy and paste relevant sections from the manuscript (include quotes in quotation marks "like this" to indicate direct quotes from your manuscript), or elaborate on this item by providing additional information not in the ms, or briefly explain why the item is not applicable/relevant for your study

"Once complete, participants were randomized to 1 of 3 study groups using a predetermined allocation list generated on Randomizer.org by a researcher not involved in the participant assignment. Participants were then assigned to a study group based on the allocation list and the order in which they were enrolled in the study."

10) Who generated the random allocation sequence, who enrolled participants, and who assigned participants to interventions

Does your paper address CONSORT subitem 10? *

Copy and paste relevant sections from the manuscript (include quotes in quotation marks "like this" to indicate direct quotes from your manuscript), or elaborate on this item by providing additional information not in the ms, or briefly explain why the item is not applicable/relevant for your study

"Once complete, participants were randomized to 1 of 3 study groups using a predetermined allocation list generated on Randomizer.org by a researcher not involved in the participant assignment. Participants were then assigned to a study group based on the allocation list and the order in which they were enrolled in the study."

11a) If done, who was blinded after assignment to interventions (for example, participants, care providers, those assessing outcomes) and how

NPT: Whether or not administering co-interventions were blinded to group assignment

11a-i) Specify who was blinded, and who wasn't

Specify who was blinded, and who wasn't. Usually, in web-based trials it is not possible to blind the participants [1, 3] (this should be clearly acknowledged), but it may be possible to blind outcome assessors, those doing data analysis or those administering co-interventions (if any).

	1	2	3	4	5	
subitem not at all important	○	○	○	○	○	essential

Does your paper address subitem 11a-i? *

Copy and paste relevant sections from the manuscript (include quotes in quotation marks "like this" to indicate direct quotes from your manuscript), or elaborate on this item by providing additional information not in the ms, or briefly explain why the item is not applicable/relevant for your study

"Participants were blinded to the other intervention protocols and did not know what intervention component was the focus of this study."

11a-ii) Discuss e.g., whether participants knew which intervention was the "intervention of interest" and which one was the "comparator"

Informed consent procedures (4a-ii) can create biases and certain expectations - discuss e.g., whether participants knew which intervention was the "intervention of interest" and which one was the "comparator".

	1	2	3	4	5	
subitem not at all important	○	○	○	○	○	essential

Does your paper address subitem 11a-ii?

Copy and paste relevant sections from the manuscript (include quotes in quotation marks "like this" to indicate direct quotes from your manuscript), or elaborate on this item by providing additional information not in the ms, or briefly explain why the item is not applicable/relevant for your study

Your answer

11b) If relevant, description of the similarity of interventions

(this item is usually not relevant for ehealth trials as it refers to similarity of a placebo or sham intervention to a active medication/intervention)

Does your paper address CONSORT subitem 11b? *

Copy and paste relevant sections from the manuscript (include quotes in quotation marks "like this" to indicate direct quotes from your manuscript), or elaborate on this item by providing additional information not in the ms, or briefly explain why the item is not applicable/relevant for your study

"Intervention
Participants were randomized into a personalized anchor (PA) group, fixed anchor (FA) group, or control group (CG). Participants in this study used their own, paid Calm accounts to access the app during the study. After completing the baseline survey, participants were sent a link to watch an instructional video that provided information about the benefits of meditating 10 minutes per day and study group–specific instructions on how to participate in the study. For those in the PA group, the video instructed participants to select an existing

in the study. For those in the PA group, the video instructed participants to select an existing routine to which they would anchor their 10 minutes of daily meditation practice. The PA group's instructional video emphasized the importance of selecting a consistently occurring daily routine that could reliably be followed by 10 or more minutes of meditation and provided clear examples of such existing routines (eg, “After I finish my coffee in the afternoon” or “After I finish breakfast in the morning”). For those in the FA group, participants were instructed to use a fixed anchor provided by the research team to which they would anchor their 10 minutes of daily meditation practice. The anchor provided was the following: “After I finish in the bathroom (brushing teeth, removing mouth guard, etc.) in the morning, I will meditate for at least 10 minutes.” Participants in the CG were given information about the mental health benefits of meditating for at least 10 minutes per day and instructed to complete 10 minutes of daily meditation but were not given any instruction on how or when to meditate. Participants were blinded to the other intervention protocols and did not know what intervention component was the focus of this study. To verify participants' comprehension of their study group–specific instructions, participants completed a 3-question comprehension quiz in Qualtrics and were given unlimited chances to answer each question correctly. Once all questions were correctly answered, participants were emailed with a start date for their intervention and they were provided with a written copy of the study instructions.

During the 8-week intervention period, all participants received a daily app–delivered reminder message (ie, push notification) to either meditate for at least 10 minutes or to meditate for at least 10 minutes using their anchor. Messages were randomly delivered at either 8 AM, 1 PM, or 6 PM (ie, a 33.3% chance of receiving the daily message at 1 of the 3 possible times), with adjustments made for participants' time zone. The message content was also randomized with a 50% chance of receiving 1 of 2 message types. The first message type included study group–specific reminders reinforcing participants' use of either their personalized or fixed anchors, or reminding the control group to meditate. The second message type was evenly randomized between reminders to use 3 motivational tools in the Calm app: mood check-ins, the meditation activity tracker, or the in-app daily reminder tool. The success of each type, timing, and sequence of daily supports was evaluated based on both participants' daily app usage data and ecological momentary assessments collected via SMS text message once per evening (8 PM) during the 8-week intervention. The results from this microrandomized trial on the effectiveness of different daily reminder messages are not reported in this paper, and it is important to note that this microrandomized trial study design meant that each message type, timing, and sequence were randomly delivered across all study groups; thus, the sequence of messages would not bias our analysis of the overall study group differences in meditation persistence during this study.

Participants were initially instructed to use their anchors (PA and FA groups) and meditate for 10 minutes per day (all groups) for 8 weeks. After 8 weeks, participants were emailed a postintervention survey to complete and were encouraged to continue meditating but were not given further instructions. Participants were emailed again at the end of the 8-week follow-up period and given a final questionnaire to complete. "

12a) Statistical methods used to compare groups for primary and secondary outcomes

NPT: When applicable, details of whether and how the clustering by care providers or centers was addressed

Does your paper address CONSORT subitem 12a? *

Copy and paste relevant sections from the manuscript (include quotes in quotation marks "like this" to indicate direct quotes from your manuscript), or elaborate on this item by providing additional information not in the ms, or briefly explain why the item is not applicable/relevant for your study

"A total sample size of 150 participants (study group sizes of 50) was targeted based on our available resources, and our expected statistical power was informed by prior interventions using the Calm app [3,55,56]. Assuming a small- to medium-effect size of 0.20, study group sizes of 50 yielded a statistical power of $1-\beta$ =.76 for detecting study group x day–level differences in linear models of our repeated daily outcome (any meditation minutes) over the 16-week study at α=.05 (calculated using GLIMMPSE [57]).
Participants' demographic, socioeconomic, and health characteristics were compared across the 3 study groups to confirm that the randomization was effective using the Kruskal-Wallis nonparametric tests of equality (Table 1).
The primary outcome measuring the odds of any daily meditation was analyzed using panel logistic regression models with participant-level random effects. Aggregate study group differences in the primary outcome were estimated using separate indicator variables for the PA and FA groups, where the CG was the omitted reference group, and differences in the primary outcome over time were estimated using interaction terms between each study group indicator variable and a daily time trend. Two modeling approaches for the daily time trend were used: (1) a single linear time trend over the full 16-week study, and (2) a piecewise linear trend with a breakpoint after the 8-week intervention (ie, daily reminder messages) being withdrawn. The same panel logistic model with random effects was estimated for an outcome variable indicating whether participants performed any minutes of meditation within 1 hour of the expected time of their anchor (referred to as "anchored meditations"). These models were estimated as intention-to-treat analyses that used daily Calm app data for all participants who were retained in the study.
In subgroup analyses, participants were split into high- and low-meditation subgroups based on their total number of days with any meditation during the 8-week intervention. The high-meditation subgroup was defined as participants who meditated on 14 (the median number of days) or more of the intervention days. All other participants were placed in the low-meditation subgroup. These subgroups were created to test whether the success of the anchoring strategy differed based on the total number of meditations performed during the intervention. Participants in the PA and FA groups were also split according to the number of intervention days that they potentially meditated with the Calm app using their anchor during the intervention. Participants from the PA and FA groups were classified as high anchorers if they completed 12 (the median number of days using one's anchor) or more meditations within 1 hour of the expected time of their anchor. All other participants in the PA and FA groups were considered low anchorers, and the CG did not use the anchoring strategy so were not classified as either high or low anchorers. These additional subgroups were created to examine how the success of the anchoring strategy varied based on the number of anchored meditations during the intervention.
Study group differences in the SRBAI between the baseline and postintervention survey were analyzed using analysis of variance (ANOVA), and pairwise comparisons between the PA and FA groups and the CG used were analyzed with the t test. All statistical analyses were performed using Stata/MP (StataCorp) 16.1 for Windows. (Microsoft Corp)"

12a-i) Imputation techniques to deal with attrition / missing values

Imputation techniques to deal with attrition / missing values: Not all participants will use the intervention/comparator as intended and attrition is typically high in ehealth trials. Specify how participants who did not use the application or dropped out from the trial were treated in the statistical analysis (a complete case analysis is strongly discouraged, and simple imputation techniques such as LOCF may also be problematic [4]).

	1	2	3	4	5	
subitem not at all important	○	○	○	○	○	essential

Does your paper address subitem 12a-i? *

Copy and paste relevant sections from the manuscript (include quotes in quotation marks "like this" to indicate direct quotes from your manuscript), or elaborate on this item by providing additional information not in the ms, or briefly explain why the item is not applicable/relevant for your study

""A total sample size of 150 participants (study group sizes of 50) was targeted based on our available resources, and our expected statistical power was informed by prior interventions using the Calm app [3,55,56]. Assuming a small- to medium-effect size of 0.20, study group sizes of 50 yielded a statistical power of 1–β =.76 for detecting study group x day–level differences in linear models of our repeated daily outcome (any meditation minutes) over the 16-week study at α=.05 (calculated using GLIMMPSE [57]).
Participants' demographic, socioeconomic, and health characteristics were compared across the 3 study groups to confirm that the randomization was effective using the Kruskal-Wallis nonparametric tests of equality (Table 1).
The primary outcome measuring the odds of any daily meditation was analyzed using panel logistic regression models with participant-level random effects. Aggregate study group differences in the primary outcome were estimated using separate indicator variables for the PA and FA groups, where the CG was the omitted reference group, and differences in the primary outcome over time were estimated using interaction terms between each study group indicator variable and a daily time trend. Two modeling approaches for the daily time trend were used: (1) a single linear time trend over the full 16-week study, and (2) a piecewise linear trend with a breakpoint after the 8-week intervention (ie, daily reminder messages) being withdrawn. The same panel logistic model with random effects was estimated for an outcome variable indicating whether participants performed any minutes of meditation within 1 hour of the expected time of their anchor (referred to as "anchored meditations"). These models were estimated as intention-to-treat analyses that used daily Calm app data for all participants who were retained in the study.
In subgroup analyses, participants were split into high- and low-meditation subgroups based on their total number of days with any meditation during the 8-week intervention. The high-meditation subgroup was defined as participants who meditated on 14 (the median number of days) or more of the intervention days. All other participants were placed in the low-meditation subgroup. These subgroups were created to test whether the success of the anchoring strategy differed based on the total number of meditations performed during the intervention. Participants in the PA and FA groups were also split according to the number of intervention days that they potentially meditated with the Calm app using their anchor during the intervention. Participants from the PA and FA groups were classified as high anchorers if they completed 12 (the median number of days using one's anchor) or more meditations within 1 hour of the expected time of their anchor. All other participants in the PA and FA groups were considered low anchorers, and the CG did not use the anchoring strategy so were not classified as either high or low anchorers. These additional subgroups were created to examine how the success of the anchoring strategy varied based on the number of anchored meditations during the intervention.
Study group differences in the SRBAI between the baseline and postintervention survey were analyzed using analysis of variance (ANOVA), and pairwise comparisons between the PA and FA groups and the CG used were analyzed with the t test. All statistical analyses were performed using Stata/MP (StataCorp) 16.1 for Windows. (Microsoft Corp)"

12b) Methods for additional analyses, such as subgroup analyses and adjusted analyses

Does your paper address CONSORT subitem 12b? *

Copy and paste relevant sections from the manuscript (include quotes in quotation marks "like this" to indicate direct quotes from your manuscript), or elaborate on this item by providing additional information not in the ms, or briefly explain why the item is not applicable/relevant for your study

"In subgroup analyses, participants were split into high- and low-meditation subgroups based on their total number of days with any meditation during the 8-week intervention. The high-meditation subgroup was defined as participants who meditated on 14 (the median number of days) or more of the intervention days. All other participants were placed in the low-meditation subgroup. These subgroups were created to test whether the success of the anchoring strategy differed based on the total number of meditations performed during the intervention. Participants in the PA and FA groups were also split according to the number of intervention days that they potentially meditated with the Calm app using their anchor during the intervention. Participants from the PA and FA groups were classified as high anchorers if they completed 12 (the median number of days using one's anchor) or more meditations within 1 hour of the expected time of their anchor. All other participants in the PA and FA groups were considered low anchorers, and the CG did not use the anchoring strategy so were not classified as either high or low anchorers. These additional subgroups were created to examine how the success of the anchoring strategy varied based on the number of anchored meditations during the intervention.
Study group differences in the SRBAI between the baseline and postintervention survey were analyzed using analysis of variance (ANOVA), and pairwise comparisons between the PA and FA groups and the CG used were analyzed with the t test. All statistical analyses were performed using Stata/MP (StataCorp) 16.1 for Windows. (Microsoft Corp)"

X26) REB/IRB Approval and Ethical Considerations [recommended as subheading under "Methods"] (not a CONSORT item)

X26-i) Comment on ethics committee approval

	1	2	3	4	5	
subitem not at all important	○	○	○	○	○	essential

Does your paper address subitem X26-i?

Copy and paste relevant sections from the manuscript (include quotes in quotation marks "like this" to indicate direct quotes from your manuscript), or elaborate on this item by providing additional information not in the ms, or briefly explain why the item is not applicable/relevant for your study

Your answer

x26-ii) Outline informed consent procedures

Outline informed consent procedures e.g., if consent was obtained offline or online (how? Checkbox, etc.?), and what information was provided (see 4a-ii). See [6] for some items to be included in informed consent documents.

	1	2	3	4	5	
subitem not at all important	○	○	○	○	○	essential

Does your paper address subitem X26-ii?

Copy and paste relevant sections from the manuscript (include quotes in quotation marks "like this" to indicate direct quotes from your manuscript), or elaborate on this item by providing additional information not in the ms, or briefly explain why the item is not applicable/relevant for your study

Your answer

X26-iii) Safety and security procedures

Safety and security procedures, incl. privacy considerations, and any steps taken to reduce the likelihood or detection of harm (e.g., education and training, availability of a hotline)

	1	2	3	4	5	
subitem not at all important	○	○	○	○	○	essential

Does your paper address subitem X26-iii?

Copy and paste relevant sections from the manuscript (include quotes in quotation marks "like this" to indicate direct quotes from your manuscript), or elaborate on this item by providing additional information not in the ms, or briefly explain why the item is not applicable/relevant for your study

Your answer

RESULTS

13a) For each group, the numbers of participants who were randomly assigned, received intended treatment, and were analysed for the primary outcome

NPT: The number of care providers or centers performing the intervention in each group and the number of patients treated by each care provider in each center

Does your paper address CONSORT subitem 13a? *

Copy and paste relevant sections from the manuscript (include quotes in quotation marks "like this" to indicate direct quotes from your manuscript), or elaborate on this item by providing additional information not in the ms, or briefly explain why the item is not applicable/relevant for your study

"A total of 2217 Calm subscribers were emailed to participate in this study. Among those who completed the eligibility survey and were identified as eligible, 168 provided informed consent, completed the baseline survey, and were randomized into 1 of the 3 study groups: (1) the PA group (n=56), (2) the FA group (n=49), or the CG (n=62). Figure 1 is a flow diagram outlining participant enrollment, randomization, and retention. "

13b) For each group, losses and exclusions after randomisation, together with reasons

Does your paper address CONSORT subitem 13b? (NOTE: Preferably, this is shown in a CONSORT flow diagram) *

Copy and paste relevant sections from the manuscript (include quotes in quotation marks "like this" to indicate direct quotes from your manuscript), or elaborate on this item by providing additional information not in the ms, or briefly explain why the item is not applicable/relevant for your study

"A total of 2217 Calm subscribers were emailed to participate in this study. Among those who completed the eligibility survey and were identified as eligible, 168 provided informed consent, completed the baseline survey, and were randomized into 1 of the 3 study groups: (1) the PA group (n=56), (2) the FA group (n=49), or the CG (n=62). Figure 1 is a flow diagram outlining participant enrollment, randomization, and retention.
After a few participants asked to withdraw (n=3), a total of 101 participants completed at least 1 postintervention survey (either week 8 or week 16) and were included in the final analysis. Due to the different attrition rates across study groups, the final analytical sample was not balanced in size across groups, limiting the statistical power of our analyses."

13b-i) Attrition diagram

Strongly recommended: An attrition diagram (e.g., proportion of participants still logging in or using the intervention/comparator in each group plotted over time, similar to a survival curve) or other figures or tables demonstrating usage/dose/engagement.

	1	2	3	4	5	
subitem not at all important	○	○	○	○	○	essential

Does your paper address subitem 13b-i?

Copy and paste relevant sections from the manuscript or cite the figure number if applicable (include quotes in quotation marks "like this" to indicate direct quotes from your manuscript), or elaborate on this item by providing additional information not in the ms, or briefly explain why the item is not applicable/relevant for your study

Your answer

14a) Dates defining the periods of recruitment and follow-up

Does your paper address CONSORT subitem 14a? *

Copy and paste relevant sections from the manuscript (include quotes in quotation marks "like this" to indicate direct quotes from your manuscript), or elaborate on this item by providing additional information not in the ms, or briefly explain why the item is not applicable/relevant for your study

"A randomized controlled trial was conducted between July 2020 and March 2021 with an 8-week intervention period, an 8-week follow-up period, and survey assessments at baseline, week 8, and week 16."

14a-i) Indicate if critical “secular events” fell into the study period

Indicate if critical “secular events” fell into the study period, e.g., significant changes in Internet resources available or “changes in computer hardware or Internet delivery resources”

	1	2	3	4	5	
subitem not at all important	○	○	○	○	○	essential

Does your paper address subitem 14a-i?

Copy and paste relevant sections from the manuscript (include quotes in quotation marks "like this" to indicate direct quotes from your manuscript), or elaborate on this item by providing additional information not in the ms, or briefly explain why the item is not applicable/relevant for your study

Your answer

14b) Why the trial ended or was stopped (early)

Does your paper address CONSORT subitem 14b? *

Copy and paste relevant sections from the manuscript (include quotes in quotation marks "like this" to indicate direct quotes from your manuscript), or elaborate on this item by providing additional information not in the ms, or briefly explain why the item is not applicable/relevant for your study

The trial was not stopped early.

15) A table showing baseline demographic and clinical characteristics for each group

NPT: When applicable, a description of care providers (case volume, qualification, expertise, etc.) and centers (volume) in each group

Does your paper address CONSORT subitem 15? *

Copy and paste relevant sections from the manuscript (include quotes in quotation marks "like this" to indicate direct quotes from your manuscript), or elaborate on this item by providing additional information not in the ms, or briefly explain why the item is not applicable/relevant for your study

Characteristic Control, n (%)
(N=37) Fixed anchor, n (%)
(N=27) Personalized anchor, n (%)
(N=37) Two-sided P valuea
Black 0 (0.00) 1 (3.70) 3 (8.11) .21
Asian/Arab 2 (5.41) 1 (3.70) 1 (2.70) .84
White 33 (89.19) 22 (81.48) 28 (75.68) .32
Bi- or multiracial 0 (0.00) 1 (3.70) 1 (2.70) .54
Race: nonresponse 2 (5.41) 0 (0.00) 2 (5.41) .47
Male 8 (21.62) 6 (22.22) 4 (10.81) .38
Female 28 (75.68) 19 (70.37) 30 (81.08) .61
Less than 20 kb 0 (0.00) 1 (3.70) 3 (8.11) .20
21-40 kb 2 (5.41) 1 (3.70) 6 (16.22) .15
41-60 kb 5 (13.51) 5 (18.52) 7 (18.92) .80
61-80 kb 2 (5.41) 2 (7.41) 1 (2.70) .69
81-100 kb 8 (21.62) 8 (29.63) 1 (2.70) .01
More than $100 kb
19 (51.35) 10 (37.04) 17 (45.95) .53
Married 26 (70.27) 12 (44.44) 17 (45.95) .05
Partnered 2 (5.41) 6 (22.22) 3 (8.11) .08
Single/divorced/widowed 9 (24.32) 9 (33.33) 17 (45.95) .15
Graduate degree 24 (64.86) 15 (55.56) 15 (40.54) .11
Bachelor's degree 6 (16.22) 10 (37.04) 12 (32.43) .14
Less than a bachelor's 7 (18.92) 2 (7.41) 10 (27.03) .14
Poor health 0 (0.00) 4 (14.81) 1 (2.70) .02
Fair health 7 (18.92) 6 (22.22) 9 (24.32) .85
Good health 12 (32.43) 10 (37.04) 13 (35.14) .93
Very good health 13 (35.14) 4 (14.81) 12 (32.43) .17
Excellent health 4 (10.81) 3 (11.11) 0 (0.00) .12
Currently with depression 11 (29.73) 9 (33.33) 11 (29.73) .94
COVID stress 30 (83.33) 20 (74.07) 27 (77.14) .66
COVID Mental Health 24 (66.67) 21 (77.78) 24 (68.57) .61
COVID Physical Health 15 (41.67) 12 (44.44) 12 (34.29) .69

15-i) Report demographics associated with digital divide issues

In ehealth trials it is particularly important to report demographics associated with digital divide issues, such as age, education, gender, social-economic status, computer/Internet/ehealth literacy of the participants, if known.

	1	2	3	4	5	
subitem not at all important	○	○	○	○	○	essential

Does your paper address subitem 15-i? *

Copy and paste relevant sections from the manuscript (include quotes in quotation marks "like this" to indicate direct quotes from your manuscript), or elaborate on this item by providing additional information not in the ms, or briefly explain why the item is not applicable/relevant for your study

Please see Table 1 for this information.

16) For each group, number of participants (denominator) included in each analysis and whether the analysis was by original assigned groups

16-i) Report multiple “denominators” and provide definitions

Report multiple “denominators” and provide definitions: Report N’s (and effect sizes) “across a range of study participation [and use] thresholds” [1], e.g., N exposed, N consented, N used more than x times, N used more than y weeks, N participants “used” the intervention/comparator at specific pre-defined time points of interest (in absolute and relative numbers per group). Always clearly define “use” of the intervention.

	1	2	3	4	5	
subitem not at all important	○	○	○	○	○	essential

Does your paper address subitem 16-i? *

Copy and paste relevant sections from the manuscript (include quotes in quotation marks "like this" to indicate direct quotes from your manuscript), or elaborate on this item by providing additional information not in the ms, or briefly explain why the item is not applicable/relevant for your study

"A total of 2217 Calm subscribers were emailed to participate in this study. Among those who completed the eligibility survey and were identified as eligible, 168 provided informed consent, completed the baseline survey, and were randomized into 1 of the 3 study groups: (1) the PA group (n=56), (2) the FA group (n=49), or the CG (n=62). Figure 1 is a flow diagram outlining participant enrollment, randomization, and retention. "

16-ii) Primary analysis should be intent-to-treat

Primary analysis should be intent-to-treat, secondary analyses could include comparing only “users”, with the appropriate caveats that this is no longer a randomized sample (see 18-i).

	1	2	3	4	5	
subitem not at all important	○	○	○	○	○	essential

Does your paper address subitem 16-ii?

Copy and paste relevant sections from the manuscript (include quotes in quotation marks "like this" to indicate direct quotes from your manuscript), or elaborate on this item by providing additional information not in the ms, or briefly explain why the item is not applicable/relevant for your study

Your answer

17a) For each primary and secondary outcome, results for each group, and the estimated effect size and its precision (such as 95% confidence interval)

Does your paper address CONSORT subitem 17a? *

Copy and paste relevant sections from the manuscript (include quotes in quotation marks "like this" to indicate direct quotes from your manuscript), or elaborate on this item by providing additional information not in the ms, or briefly explain why the item is not applicable/relevant for your study

"To examine if the anchoring strategy was more successful for more frequent meditators, Figure 3 displays the raw and predicted daily probability of any minutes of meditation among the high-meditation subgroup (n=51) in each study group. The corresponding regression results in Table 2 show that among the high-meditation subgroup, the FA group still had a significantly higher average odds of daily meditation during the intervention (1.08 OR; 95% CI 1.02-1.31; P=.03), and all participants experienced a significant linear decline in their odds of daily meditation meditating during the 8-week intervention (0.96 OR; 95% CI 0.96-0.97; P<.001). However, there was no statistically significant difference between study groups in the decline of daily odds of meditation during the 8-week follow-up among the high-meditation subgroup."

17a-i) Presentation of process outcomes such as metrics of use and intensity of use

In addition to primary/secondary (clinical) outcomes, the presentation of process outcomes such as metrics of use and intensity of use (dose, exposure) and their operational definitions is critical. This does not only refer to metrics of attrition (13-b) (often a binary variable), but also to more continuous exposure metrics such as “average session length”. These must be accompanied by a technical description how a metric like a “session” is defined (e.g., timeout after idle time) [1] (report under item 6a).

	1	2	3	4	5	
subitem not at all important	○	○	○	○	○	essential

Does your paper address subitem 17a-i?

Copy and paste relevant sections from the manuscript (include quotes in quotation marks "like this" to indicate direct quotes from your manuscript), or elaborate on this item by providing additional information not in the ms, or briefly explain why the item is not applicable/relevant for your study

Your answer

17b) For binary outcomes, presentation of both absolute and relative effect sizes is recommended

Does your paper address CONSORT subitem 17b? *

Copy and paste relevant sections from the manuscript (include quotes in quotation marks "like this" to indicate direct quotes from your manuscript), or elaborate on this item by providing additional information not in the ms, or briefly explain why the item is not applicable/relevant for your study

"To examine if the anchoring strategy was more successful for more frequent meditators, Figure 3 displays the raw and predicted daily probability of any minutes of meditation among the high-meditation subgroup (n=51) in each study group. The corresponding regression results in Table 2 show that among the high-meditation subgroup, the FA group still had a significantly higher average odds of daily meditation during the intervention (1.08 OR; 95% CI 1.02-1.31; P=.03), and all participants experienced a significant linear decline in their odds of daily meditation meditating during the 8-week intervention (0.96 OR; 95% CI 0.96-0.97; P<.001). However, there was no statistically significant difference between study groups in the decline of daily odds of meditation during the 8-week follow-up among the high-meditation subgroup."

18) Results of any other analyses performed, including subgroup analyses and adjusted analyses, distinguishing pre-specified from exploratory

Does your paper address CONSORT subitem 18? *

Copy and paste relevant sections from the manuscript (include quotes in quotation marks "like this" to indicate direct quotes from your manuscript), or elaborate on this item by providing additional information not in the ms, or briefly explain why the item is not applicable/relevant for your study

"To visualize how the anchoring strategy impacted meditation persistence, Figure 4 plots the average daily percent of participants who completed any minutes of meditation within 1 hour of the expected time of their anchor (ie, anchored meditations) among the high anchorers (n=19) and separately among the low anchorers (n=45). The high-anchorer subgroup was composed of 13 participants from the FA group and 6 participants from the PA group, which demonstrates the relative success of using the fixed morning anchor versus allowing participants to select their own anchor. Additionally, 4 out of the 6 high anchorers from the PA group selected a morning anchor that occurred between 7 AM and 9 AM, which further suggests that morning anchors are the most likely to be successful. The trends in Figure 4 show that most participants (ie, the low anchorers) did not use their anchoring strategy beyond the first 4 weeks of the intervention but that anchored meditations remained fairly persistent among the high anchorers.
The stronger persistence in anchored meditations among the high anchorers was tested empirically and is shown in Table 3, which displays the panel logistic regression results from models predicting the odds of any minutes of meditation for the low anchorers and those in the CG or the odds of any anchored meditations among the high anchorers. This split outcome variable provided a more conservative test of the differences in meditation persistence between the high anchorers versus the low anchorers or the CG because all nonanchored meditations were not considered as evidence of meditation persistence for the high anchorers. The high anchorers had a significantly higher average odds of daily meditation during the intervention (34.68 OR; 95% CI 5.70-210.80; P=.008), and all participants experienced a significant linear decline in their odds of daily meditation during the 8-week intervention (0.96 OR; 95% CI 0.96-0.97; P<.001). Importantly, the high anchorers showed a significantly smaller decline in the linear trend of their odds of daily meditation during the 8-week follow-up period (their daily trend increased by 1.13 OR from their trend during the intervention; 95% CI 1.02-1.35; P=.007 during the follow-up). A separate model was estimated for this split outcome that also included measures of participants' race, gender, education, marital status, health status, and an identifier for self-reporting being depressed, and these results are presented in Multimedia Appendix 1 and do not significantly differ from the model without these additional participant characteristics.
Figure 5 displays the raw and predicted probability of this split outcome for high anchorers, low anchorers, and the CG. "

18-i) Subgroup analysis of comparing only users

A subgroup analysis of comparing only users is not uncommon in ehealth trials, but if done, it must be stressed that this is a self-selected sample and no longer an unbiased sample from a randomized trial (see 16-iii).

	1	2	3	4	5	
subitem not at all important	○	○	○	○	○	essential

Does your paper address subitem 18-i?

Copy and paste relevant sections from the manuscript (include quotes in quotation marks "like this" to indicate direct quotes from your manuscript), or elaborate on this item by providing additional information not in the ms, or briefly explain why the item is not applicable/relevant for your study

Your answer

19) All important harms or unintended effects in each group

(for specific guidance see CONSORT for harms)

Does your paper address CONSORT subitem 19? *

Copy and paste relevant sections from the manuscript (include quotes in quotation marks "like this" to indicate direct quotes from your manuscript), or elaborate on this item by providing additional information not in the ms, or briefly explain why the item is not applicable/relevant for your study

There were no harms or unintended effects.

19-i) Include privacy breaches, technical problems

Include privacy breaches, technical problems. This does not only include physical “harm” to participants, but also incidents such as perceived or real privacy breaches [1], technical problems, and other unexpected/unintended incidents. “Unintended effects” also includes unintended positive effects [2].

	1	2	3	4	5	
subitem not at all important	O	O	O	O	O	essential

Does your paper address subitem 19-i?

Copy and paste relevant sections from the manuscript (include quotes in quotation marks "like this" to indicate direct quotes from your manuscript), or elaborate on this item by providing additional information not in the ms, or briefly explain why the item is not applicable/relevant for your study

Your answer

19-ii) Include qualitative feedback from participants or observations from staff/researchers

Include qualitative feedback from participants or observations from staff/researchers, if available, on strengths and shortcomings of the application, especially if they point to unintended/unexpected effects or uses. This includes (if available) reasons for why people did or did not use the application as intended by the developers.

	1	2	3	4	5	
subitem not at all important	O	O	O	O	O	essential

Does your paper address subitem 19-ii?

Copy and paste relevant sections from the manuscript (include quotes in quotation marks "like this" to indicate direct quotes from your manuscript), or elaborate on this item by providing additional information not in the ms, or briefly explain why the item is not applicable/relevant for your study

Your answer

DISCUSSION

22) Interpretation consistent with results, balancing benefits and harms, and considering other relevant evidence

NPT: In addition, take into account the choice of the comparator, lack of or partial blinding, and unequal expertise of care providers or centers in each group

22-i) Restate study questions and summarize the answers suggested by the data, starting with primary outcomes and process outcomes (use)

Restate study questions and summarize the answers suggested by the data, starting with primary outcomes and process outcomes (use).

	1	2	3	4	5	
subitem not at all important	○	○	○	○	○	essential

Does your paper address subitem 22-i? *

Copy and paste relevant sections from the manuscript (include quotes in quotation marks "like this" to indicate direct quotes from your manuscript), or elaborate on this item by providing additional information not in the ms, or briefly explain why the item is not applicable/relevant for your study

"This study tested the efficacy of using either PAs or FAs for establishing a persistent meditation app routine with the mobile app Calm. Although the results found that all study groups (ie, PA, FA, and CG) experienced an equal decline in their daily odds of performing any minutes of meditation with the Calm app during the 8-week intervention, the FA group was significantly more persistent (ie, smaller daily decline in the odds of any meditation) during the 8-week follow-up period. Subgroup analyses revealed that performing a larger number of meditations during the intervention was not sufficient for displaying meditation persistence. Instead, the participants who were high anchorers during the intervention (ie, equal to or above the median number of meditations performed within 1 hour of the expected time of their anchor) showed the most persistent meditation routines during the follow-up period. These findings indicate that the anchoring strategy can create persistent meditation routines for some participants but that additional intervention tools are likely needed to help more participants successfully adhere to their anchored meditation routine. "

22-ii) Highlight unanswered new questions, suggest future research

Highlight unanswered new questions, suggest future research.

	1	2	3	4	5	
subitem not at all important	○	○	○	○	○	essential

Does your paper address subitem 22-ii?

Copy and paste relevant sections from the manuscript (include quotes in quotation marks "like this" to indicate direct quotes from your manuscript), or elaborate on this item by providing additional information not in the ms, or briefly explain why the item is not applicable/relevant for your study

Your answer

20) Trial limitations, addressing sources of potential bias, imprecision, and, if relevant, multiplicity of analyses

20-i) Typical limitations in ehealth trials

Typical limitations in ehealth trials: Participants in ehealth trials are rarely blinded. Ehealth trials often look at a multiplicity of outcomes, increasing risk for a Type I error. Discuss biases due to non-use of the intervention/usability issues, biases through informed consent procedures, unexpected events.

	1	2	3	4	5	
subitem not at all important	○	○	○	○	○	essential

Does your paper address subitem 20-i? *

Copy and paste relevant sections from the manuscript (include quotes in quotation marks "like this" to indicate direct quotes from your manuscript), or elaborate on this item by providing additional information not in the ms, or briefly explain why the item is not applicable/relevant for your study

"Although this was the first study to use personalized or fixed anchors for establishing a persistent meditation app routine with a consumer-based app (ie, Calm) and there were no unexpected events, there were still a number of limitations. First, we had a homogeneous, small sample size limiting the generalizability of our findings, particularly to other racial groups and people of different socioeconomic status. Second, our study targeted dormant users of Calm who had paid for an annual subscription but had not recently used the app, which again limits the generalizability of our results for other types of app users. Third, the daily app–delivered reminder messages appeared to be an ineffective method of boosting most participants' attention to and use of the anchoring strategy, so it is difficult to know whether a longer duration of intervention or increased intervention supports are necessary to increase adherence to the anchoring strategy and more rigorously test the efficacy of this intervention approach for establishing behavioral routines. Finally, a significant degree of study attrition from either withdrawals or missing survey data occurred during the intervention, which limited the statistical power of our analyses. "

21) Generalisability (external validity, applicability) of the trial findings

NPT: External validity of the trial findings according to the intervention, comparators, patients, and care providers or centers involved in the trial

21-i) Generalizability to other populations

Generalizability to other populations: In particular, discuss generalizability to a general Internet population, outside of a RCT setting, and general patient population, including applicability of the study results for other organizations

	1	2	3	4	5	
subitem not at all important	O	O	O	O	O	essential

Does your paper address subitem 21-i?

Copy and paste relevant sections from the manuscript (include quotes in quotation marks "like this" to indicate direct quotes from your manuscript), or elaborate on this item by providing additional information not in the ms, or briefly explain why the item is not applicable/relevant for your study

Your answer

21-ii) Discuss if there were elements in the RCT that would be different in a routine application setting

Discuss if there were elements in the RCT that would be different in a routine application setting (e.g., prompts/reminders, more human involvement, training sessions or other co-interventions) and what impact the omission of these elements could have on use, adoption, or outcomes if the intervention is applied outside of a RCT setting.

	1	2	3	4	5	
subitem not at all important	O	O	O	O	O	essential

Does your paper address subitem 21-ii?

Copy and paste relevant sections from the manuscript (include quotes in quotation marks "like this" to indicate direct quotes from your manuscript), or elaborate on this item by providing additional information not in the ms, or briefly explain why the item is not applicable/relevant for your study

Your answer

OTHER INFORMATION

23) Registration number and name of trial registry

Does your paper address CONSORT subitem 23? *

Copy and paste relevant sections from the manuscript (include quotes in quotation marks "like this" to indicate direct quotes from your manuscript), or elaborate on this item by providing additional information not in the ms, or briefly explain why the item is not applicable/relevant for your study

Mindfulness Takes Practice NCT04378530

24) Where the full trial protocol can be accessed, if available

Does your paper address CONSORT subitem 24? *

Cite a Multimedia Appendix, other reference, or copy and paste relevant sections from the manuscript (include quotes in quotation marks "like this" to indicate direct quotes from your manuscript), or elaborate on this item by providing additional information not in the ms, or briefly explain why the item is not applicable/relevant for your study

The full trial protocol can be accessed in the paper.

25) Sources of funding and other support (such as supply of drugs), role of funders

Does your paper address CONSORT subitem 25? *

Copy and paste relevant sections from the manuscript (include quotes in quotation marks "like this" to indicate direct quotes from your manuscript), or elaborate on this item by providing additional information not in the ms, or briefly explain why the item is not applicable/relevant for your study

"funded by Arizona State University"

X27) Conflicts of Interest (not a CONSORT item)

X27-i) State the relation of the study team towards the system being evaluated

In addition to the usual declaration of interests (financial or otherwise), also state the relation of the study team towards the system being evaluated, i.e., state if the authors/evaluators are distinct from or identical with the developers/sponsors of the intervention.

	1	2	3	4	5	
subitem not at all important	○	○	○	○	○	essential

Does your paper address subitem X27-i?

Copy and paste relevant sections from the manuscript (include quotes in quotation marks "like this" to indicate direct quotes from your manuscript), or elaborate on this item by providing additional information not in the ms, or briefly explain why the item is not applicable/relevant for your study

Your answer

About the CONSORT EHEALTH checklist

As a result of using this checklist, did you make changes in your manuscript? *

- ○ yes, major changes
- ○ yes, minor changes
- ◉ no

What were the most important changes you made as a result of using this checklist?

Your answer

How much time did you spend on going through the checklist INCLUDING making changes in your manuscript *

Going through the checklist took over 5 hours.

As a result of using this checklist, do you think your manuscript has improved? *

- ( ) yes
- (x) no
- ( ) Other:

Would you like to become involved in the CONSORT EHEALTH group?

This would involve for example becoming involved in participating in a workshop and writing an "Explanation and Elaboration" document

- ( ) yes
- ( ) no
- ( ) Other:

Any other comments or questions on CONSORT EHEALTH

Your answer

STOP - Save this form as PDF before you click submit

To generate a record that you filled in this form, we recommend to generate a PDF of this page (on a Mac, simply select "print" and then select "print as PDF") before you submit it.

When you submit your (revised) paper to JMIR, please upload the PDF as supplementary file.

Don't worry if some text in the textboxes is cut off, as we still have the complete information in our database. Thank you!

Final step: Click submit !

Click submit so we have your answers in our database!

Submit Clear form

Never submit passwords through Google Forms.

This form was created outside of your domain. Report Abuse - Terms of Service - Privacy Policy

Google Forms
