# Supplementary material for: Using Personalized Anchors to Establish Routine Meditation Practice With a Mobile App: Randomized Controlled Trial
Source: JMIR Mhealth Uhealth. 2021 Dec 22;9(12):e32794. doi: 10.2196/32794 (PMC8734923; doi:10.2196/32794)
Supplement: Multimedia Appendix 2 [file mhealth_v9i12e32794_app2.docx]

**Table A1: Treatment Effects on the Probability of Daily Meditation**

|  | (1) | (2) |
| --- | --- | --- |
|  | All Participants | High Meditators |
| Fixed anchor | 1.188^**^ | 1.048^*^ |
|  | [1.004,1.406] | [0.983,1.352] |
| Personalized anchor | 1.033 | 1.111 |
|  | [0.884,1.208] | [0.882,1.398] |
| Days in study | 0.995^***^ | 0.993^***^ |
|  | [0.994,0.996] | [0.992,0.995] |
| Fixed anchor x Days | 0.989 | 1.001 |
|  | [0.977,1.000] | [0.999,1.003] |
| Personalized anchor x Days | 0.993 | 0.987^*^ |
|  | [0.976,1.002] | [0.974,1.000] |
| Days post-intervention | 0.992 | 0.985^*^ |
|  | [0.978,1.006] | [0.969,1.000] |
| Fixed anchor x Post-int. Days | 1.035^***^ | 1.005 |
|  | [1.013,1.057] | [0.978,1.032] |
| Personalized anchor x Post-int. Days | 1.013 | 0.985^*^ |
|  | [0.990,1.036] | [0.975,1.000] |
| White | *Reference* | |
|  |  |  |
| Black | 1.407^*^ | 1.054 |
|  | [0.966,2.049] | [0.677,1.640] |
| Asian | 1.033 | 1.772^*^ |
|  | [0.706,1.512] | [0.911,3.448] |
| Biracial | 0.834 | 0.664 |
|  | [0.466,1.494] | [0.364,1.210] |
| Race non-response | 0.864 | 1.001 |
|  | [0.600,1.245] | [0.525,1.905] |
| Male | *Reference* | |
|  |  |  |
| Female | 1.003 | 1.101 |
|  | [0.864,1.165] | [0.900,1.347] |
| Income >$100k | *Reference* | |
|  |  |  |
| Income <$20k | 0.972 | 1.168 |
|  | [0.691,1.368] | [0.698,1.955] |
| Income $21k - $40k | 0.868 | 1.510 |
|  | [0.656,1.147] | [0.841,2.714] |
| Income $41k - $60k | 0.893 | 0.955 |
|  | [0.732,1.090] | [0.711,1.283] |
| Income $61k - $80k | 0.930 | 1.165 |
|  | [0.689,1.257] | [0.701,1.937] |
| Income $81k - $100k | 0.917 | 0.916 |
|  | [0.764,1.101] | [0.707,1.186] |
| Single | *Reference* | |
| Married | 1.001 | 1.206 |
|  | [0.830,1.208] | [0.903,1.611] |
| Partnered | 0.901 | 1.023 |
|  | [0.704,1.153] | [0.637,1.642] |
| Less than graduate degree | *Reference* | |
|  |  |  |
| Graduate degree | 0.957 | 1.027 |
|  | [0.836,1.096] | [0.830,1.271] |
| Excellent health | *Reference* | |
|  |  |  |
| Poor health | 0.684^**^ | 1.000 |
|  | [0.468,0.999] | [1.000,1.000] |
| Fair health | 0.835 | 0.714 |
|  | [0.633,1.102] | [0.478,1.068] |
| Good health | 0.879 | 0.762 |
|  | [0.678,1.138] | [0.519,1.118] |
| Very good health | 0.866 | 0.709^*^ |
|  | [0.665,1.128] | [0.483,1.040] |
| Depression | 0.931 | 1.106 |
|  | [0.803,1.079] | [0.871,1.406] |
| **Participant-day observations** | **11,312** | **5,712** |
| **Participants** | **101** | **51** |

**Note**: This table presents the exponentiated coefficients (odds ratios) from panel logistic regressions estimated with participant-level random effects predicting a binary outcome equal to one if the participant meditated using the Calm app on a given day (95% confidence intervals are in brackets). Column (1) shows the regression results from the full sample and column (2) shows the results estimated over the subsample of participants with above the median number of days with any meditations during the eight-week intervention (median = 14 days with any meditation).

^*^ *p* < 0.10, ^**^ *p* < 0.05, ^***^ *p* < 0.01

**Table A2: Effect of Successfully Anchoring on the Probability of Daily Meditation**

|  | (1) | (2) |
| --- | --- | --- |
| <12 Cued Meditations | 0.418 | 0.544 |
|  | [0.138,1.267] | [0.177,1.672] |
| >=12 Cued Meditations | 34.955^***^ | 43.049^***^ |
|  | [6.595,185.283] | [7.833,236.581] |
| Days in study | 0.960^***^ | 0.964^***^ |
|  | [0.957,0.964] | [0.957,0.971] |
| <12 Cued x Days | 0.998 | 0.987 |
|  | [0.993,1.004] | [0.977,1.002] |
| >=12 Cued x Days | 1.002 | 0.994 |
|  | [0.994,1.010] | [0.979,1.009] |
| Days post-intervention |  | 0.992 |
|  |  | [0.978,1.006] |
| <12 Cued x Post-int. Days |  | 1.017 |
|  |  | [0.990,1.045] |
| >=12 Cued x Post-int. Days |  | 1.029^***^ |
|  |  | [1.009,1.050] |
| White | *Reference* | |
|  |  |  |
| Black | 125.879^***^ | 119.395^***^ |
|  | [5.338,2968.246] | [5.122,2783.198] |
| Asian | 0.170 | 0.173 |
|  | [0.006,4.640] | [0.006,4.669] |
| Biracial | 1.148 | 1.171 |
|  | [0.021,64.075] | [0.021,64.487] |
| Race non-response | 0.188 | 0.189 |
|  | [0.014,2.578] | [0.014,2.560] |
| Male | *Reference* | |
|  |  |  |
| Female | 1.417 | 1.395 |
|  | [0.461,4.360] | [0.455,4.274] |
| Income >$100k | *Reference* | |
|  |  |  |
| Income <$20k | 1.380 | 1.392 |
|  | [0.128,14.901] | [0.130,14.914] |
| Income $21k - $40k | 0.159^*^ | 0.157^*^ |
|  | [0.021,1.225] | [0.020,1.206] |
| Income $41k - $60k | 0.328 | 0.332 |
|  | [0.075,1.423] | [0.077,1.436] |
| Income $61k - $80k | 2.403 | 2.391 |
|  | [0.212,27.257] | [0.213,26.898] |
| Income $81k - $100k | 0.280^*^ | 0.284^*^ |
|  | [0.075,1.049] | [0.076,1.059] |
| Single | *Reference* | |
|  |  |  |
| Married | 0.590 | 0.595 |
|  | [0.155,2.255] | [0.156,2.263] |
| Partnered | 0.206^*^ | 0.208^*^ |
|  | [0.036,1.190] | [0.036,1.195] |
| Less than graduate degree | *Reference* | |
|  |  |  |
| Graduate degree | 0.513 | 0.511 |
|  | [0.184,1.431] | [0.184,1.422] |
| Excellent health | *Reference* | |
|  |  |  |
| Poor health | 0.123 | 0.122 |
|  | [0.007,2.057] | [0.007,2.013] |
| Fair health | 0.285 | 0.281 |
|  | [0.031,2.637] | [0.031,2.579] |
| Good health | 0.169^*^ | 0.167^*^ |
|  | [0.022,1.277] | [0.022,1.253] |
| Very good health | 0.135^*^ | 0.135^*^ |
|  | [0.017,1.042] | [0.018,1.032] |
| Depression | 0.699 | 0.700 |
|  | [0.242,2.019] | [0.243,2.014] |
| **Participant-day observations** | **11,312** | **5,712** |
| **Participants** | **101** | **51** |

**Note**: This table presents the exponentiated coefficients (odds ratios) from panel logistic regressions estimated with participant-level random effects predicting a binary outcome equal to one if the participant meditated using the Calm app on a given day for the control group and those with <12 anchored meditations during the intervention and equal to one if the participant meditated according to their anchor among those with ≥12 anchored meditations during the intervention (95% confidence intervals are in brackets). Column (1) shows the regression results when using a single linear time trend, and column (2) shows the regression results when estimating separate linear trends before and after the intervention ended on day 56 (end of week 8). ^*^ *p* < 0.10, ^**^ *p* < 0.05, ^***^ *p* < 0.01
